# Establishment survey participation during the COVID-19 pandemic

**DOI:** 10.1186/s12651-022-00321-8

**Published:** 2022-11-13

**Authors:** Benjamin Küfner, Joseph W. Sakshaug, Stefan Zins

**Affiliations:** 1 Institute for Employment Research, Regensburgerstr. 100, 90478 Nuremberg, Germany; 2 University of Warwick, CV4 7AL Coventry, UK; 3 Ludwig Maximilian University of Munich, Ludwigstr. 33, 80539 Munich, Germany

**Keywords:** Fieldwork effort, Nonresponse bias, Data collection, Weighting adjustment administrative data, IAB Job Vacancy Survey, C83

## Abstract

Establishment surveys around the globe have measured the impact of the COVID-19 pandemic on establishments’ conditions and business practices. At the same time, the consequences of the pandemic, such as closures, hygiene standards, or remote work arrangements, may have also altered patterns of survey participation and introduced nonresponse bias, threatening the quality of establishment survey data. To investigate these issues, this article examines fieldwork outcomes, nonresponse bias, and predictors of survey participation in the IAB-Job Vacancy Survey. As comparisons with previous survey years show, it became more difficult to successfully interview establishments during the COVID-19 pandemic. Using linked administrative data, we show that nonresponse bias was higher in 2020 compared to previous years, even after applying the standard weighting adjustment. However, general patterns of survey participation in 2020 were similar to previous years and COVID-19 related measures were not strong predictors of survey participation in 2020. Further, we provide evidence that nonresponse bias during the pandemic can be reduced by incorporating additional administrative variables into the weighting procedure relative to the standard weighting variables. We conclude this article with a discussion of the findings and implications for survey practitioners.

**Supplementary Information:**

The online version contains supplementary material available at 10.1186/s12651-022-00321-8.

## Introduction

In 2020, the COVID-19 pandemic hit the world and not only changed the lives of many individuals, but also forced establishments to adjust their behavior. Establishments were severely affected by lockdowns, containment regulations, home office recommendations, and unprecedented supply and demand shocks. All of these aspects led to changes in operational activities, the introduction of new organizational routines, or the (temporal) closure of entire establishments (e.g. Brinca et al. 2020; Donthu and Gustafsson 2020).

Especially in these dynamic times, establishment survey data are used to form the basis for policy decisions and are heavily used in scientific research. The IAB-Job Vacancy Survey (IAB-JVS) is one example of an ongoing establishment survey that was fielded and analyzed for policy briefing publications during the COVID-19 pandemic (e.g. Gürtzgen and Kubis 2021). This voluntary and nationwide establishment survey captures the number and structure of job vacancies, as well as detailed information about recruiting processes from up to 14,000 respondents in Germany each year (Bossler et al. 2020). In 2020, it also covered various items on COVID-19 including layoffs due to the pandemic and receipt of government subsidies.

It is reasonable to expect that the COVID-19 pandemic not only affected substantive measures, but also participation in voluntary establishment surveys. For instance, closure of entire establishments or reduced working hours might negatively affect the ability of establishments to take part in a survey. Some establishments had to develop new processes for handling survey requests as their pre-pandemic routines couldn’t be executed. However, there are also reasons to expect that the pandemic might have had a positive impact on survey participation, for example, establishments which are dealing with smaller workloads may have more availability to respond to survey requests. There may also be greater motivation to express their opinions about the crisis and the government-imposed regulations. In addition, establishments receiving government benefits (e.g. short-time work subsidies) might feel more obligated to participate in a government-sponsored survey.

If the pandemic affected the survey participation behaviour of establishments, then nonresponse bias might also be affected, perhaps more so compared to previous years. Nonresponse bias adversely impacts data quality and, hence, threatens the validity of substantive analyses of economic indicators and substantive phenomena derived from the collected data (e.g. Lineback and Thompson 2010; Thompson and Oliver 2012). For this reason, investigations of nonresponse bias are important for assessing the quality of establishment survey data (e.g. Lineback and Thompson 2010; Earp et al. 2018; König et al. 2021; Küfner et al. 2022).^1^

This study analyzes survey participation and nonresponse bias in the IAB-JVS during the COVID-19 pandemic by accomplishing four research objectives. First, we analyze fieldwork effort and survey participation outcomes (response rates, contact rates, cooperation rates) in the three quarterly reinterviews of the IAB-JVS that were affected by the COVID-19 pandemic in 2020 compared to the two preceding (non-pandemic) years of 2019 and 2018. Second, we examine the magnitude of nonresponse bias in 2020 compared to the preceding years of 2019 and 2018. Nonresponse bias is assessed for multiple variables, including the COVID-19 short-time work subsidy (or “Kurzarbeit”)^2^. Third, we examine whether COVID-19 related measures, such as short-time work, containment regulations, regional information about COVID-19 incidence rates, and mobility reduction were strong predictors of establishment survey participation, and whether general predictors of survey participation changed in 2020 compared to previous years. Fourth, we evaluate the performance of the current IAB-JVS weighting adjustment scheme relative to a new weighting adjustment scheme based on extensive administrative data for reducing nonresponse bias during the pandemic year of 2020 compared to previous years. Additionally, we test whether nonresponse bias adjustments in 2020 could be improved by including COVID-19 related auxiliary variables in the weighting procedure.

In summary, this study addresses the following research questions:

- RQ1: *Did fieldwork and participation outcomes in the IAB-Job Vacancy Survey differ in 2020 (i.e. the COVID-19 year) compared to previous years?*
- RQ2: *Did nonresponse bias differ in 2020 (i.e. the COVID-19 year) compared to previous years? Was the key survey variable, receipt of short-time work benefits, affected by nonresponse bias?*
- RQ3: *To what extent did survey participation patterns differ during the COVID-19 pandemic compared to previous years? Are COVID-19 related variables strong predictors of establishment survey participation in 2020?*
- RQ4: *To what extent do nonresponse adjustment weights reduce nonresponse bias in 2020 (i.e. the COVID-19 year) compared to previous years? Does the performance of the weights improve by incorporating additional administrative and COVID-19 related variables into the weighting scheme?*

Answers to these questions are not only important for the IAB-JVS, but also for establishment surveys in general. With this analysis, we contribute to the literature by providing evidence on whether establishment survey participation and the composition of respondents changed during the COVID-19 pandemic. This study also provides indications on whether establishment surveys may benefit from revising their weighting scheme to include additional auxiliary variables (e.g. COVID-19 specific) to counteract the risk of nonresponse bias. In addition, the proposed analysis may serve as a blueprint for other establishment surveys on how to evaluate and address nonresponse bias during the COVID-19 pandemic.

The balance of this article is structured as follows. In Sect. 2 , we briefly review the relevant literature related to the research aims. Section 3 describes the used data sources and proposed methods. The results are presented in Sect. 4 . In Sect. 5 the findings of the study and their practical implications for survey researchers are discussed and the results are summarized in Sect. 6.

## Background

### The COVID-19 pandemic and establishment survey participation

The COVID-19 pandemic had a clear effect on establishment survey participation as evidenced by response rates worldwide. Strong decreases in response rates were observed in Spring 2020 for many voluntary surveys administered by the US Bureau of Labour Statistics (BLS), including the Job Openings and Labour Turnover Survey (up to ten percentage points), the Consumer Price Index (up to sixteen percentage points), and the Employment Cost Index (up to five percentage points). However, other BLS surveys, mostly mandatory ones, have been able to maintain stable response rates, including the Annual Refilling Survey (U.S. Bureau of Labor Statistics 2021). The US Business Uncertainty Panel, a voluntary survey, experienced a decreased response rate (about eight percentage points) during the beginning of the COVID-19 pandemic (Altig et al. 2020). Kožuh (2021) reports that response rates to short time statistics in Slovenia, especially in March, April, and May 2020, were negatively affected by the pandemic; for example, in the mandatory Short Time Statistics for Output Prices response rates dropped by up to seven percentage points. Similarly, response rates to several business surveys at the National Statistical Office of Portugal dropped by close to ten percentage points (Moreira et al. 2021).

There is limited evidence on response rates for establishment subgroups during the pandemic. The mandatory Annual Enterprise Survey from New Zealand noticed that industries strongly affected by lockdown activities, such as accommodation, food and beverage, and travel agency services had lower response rates than in previous years (McKenzie 2021). Kagerl et al. (2022) showed that establishments receiving short-time work subsidies were more likely to participate in BeCovid, a voluntary establishment survey in Germany. The response rate was up to 8 percentage points higher for establishments with long periods of short-time work than for establishments without short-time work. Investigating response rates in the US COVID-19 Business Impacts Study showed that participation was not differentially affected by establishments’ revenue or industry type, but small businesses with fewer than 21 employees were more likely to participate than larger businesses (Stapleton et al. 2021). The latter finding is, however, typical of voluntary establishment surveys and may be driven by other factors unrelated to the pandemic (e.g. Earp et al. 2018; Hecht et al. 2019; König et al. 2021; Küfner et al. 2022).

Not only were response rates likely affected by the COVID-19 crises, but also the fieldwork effort that was necessary to contact and recruit survey participants. Evidence from the US Health and Retirement Study, a voluntary household survey, suggests that call attempts were not as productive as in previous years, resulting in more call attempts per completed interview (Weir 2020). This was also likely true for establishment surveys where reduced working hours and (temporary) establishment closures may have contributed to lower contact rates and more call attempts necessary to reach establishments. Even among those establishments that could be reached some might have refused to participate for similar reasons (e.g. the target respondent was released from work or on short-time work). On the other hand, some establishments that were contacted may have been more motivated to cooperate in order to express their views about the pandemic or out of a perceived sense of obligation, especially if they received government subsidies. Thus, cooperation (or refusal) rates were also likely affected by the pandemic.

### Nonresponse bias in establishment surveys

To analyze nonresponse bias, researchers often compare respondents and nonrespondents using auxiliary information available for both groups (Lineback and Thompson 2010). As illustrated in previous research (Janik and Kohaut 2012; König et al. 2021; Küfner et al. 2022; Rothbaum et al. 2021), extensive administrative data are a promising source of auxiliary information for studying nonresponse bias that overcomes the limitations of other auxiliary sources, such as sampling frame data, paradata, or comparisons between early and late respondents.

The literature has documented several examples of nonresponse bias in establishment surveys. The most prominent example is establishment size. Most studies find that larger establishments are less likely to participate in surveys than smaller ones (Janik and Kohaut 2012; Earp et al. 2018; Hecht et al. 2019; König et al. 2021; Küfner et al. 2022), with some exceptions for surveys that implement special treatments on larger establishments, including adaptive recruitment strategies (Davis and Pihama 2009; Seiler 2014). Nonresponse biases are also found for industry type (Tomaskovic-Devey et al. 1995), survey topic (HMRC 2010; Snijkers et al. 2013; Snijkers 2018; Küfner et al. 2022), establishment age (Phipps and Jones 2007), average wages (Phipps et al. 2012; Küfner et al. 2022), and region of the establishment (Janik 2011; König et al. 2021), among others.

The COVID-19 pandemic may have introduced new nonresponse biases or altered existing ones. Thereby, the effect of the COVID-19 pandemic on nonresponse bias could be two-fold: (1) New biases could arise due to the differential impact that the pandemic and the government’s mitigation efforts had on specific types of establishments. For instance, establishments that receive short-time work benefits may be more (or less) likely to participate compared to establishments who do not receive this subsidy; and (2) Nonresponse biases that were robust until 2020 could be altered.

To our knowledge, no comprehensive analysis of nonresponse bias in establishment surveys has been carried out in the context of the COVID-19 pandemic. Given the importance of collecting high-quality data from establishments and the enhanced risk of nonresponse bias during the pandemic, such an analysis would fill a timely research gap.

### Auxiliary variables for nonresponse weighting adjustments

Weighting adjustments are typically used to counter nonresponse bias in establishment surveys. One such approach is response propensity weighting (Valliant et al. 2013). As Little and Vartivarian (2005) show, the effectiveness of weighting depends on the availability and quality of auxiliary data. Auxiliary variables that are highly correlated with both the participation outcome and the survey variables of interest are most effective at reducing nonresponse bias. However, the availability of extensive auxiliary information for respondents and nonrespondents in establishment surveys is rare and often limited to a small set of sampling frame variables or paradata that may correlate with participation but not necessarily with the substantive survey variables. As shown by Küfner et al. (2022), administrative data are a promising source for auxiliary variables and can help to reduce nonresponse bias in establishment surveys generally. But there is also reason to believe that such data can help to reduce nonresponse bias during the COVID-19 pandemic. For example, administrative variables about establishment subsidies, such as the receipt of short-time work benefits, are promising candidates for use in nonresponse bias adjustments as they are likely correlated with both participation and the substantive survey variables. The benefits of using administrative data in weighting adjustments during the COVID-19 crises are also reflected in the empirical analysis of Rothbaum et al. (2021). They showed that a revised weighting strategy using entropy balancing based on extensive administrative data reduced nonresponse bias in the American Community Survey, a mandatory household panel, compared to the standard weighting approach.

In addition, regional-level indicators of the pandemic may be a useful source of auxiliary information for nonresponse adjustments. Regional COVID-19 outbreaks have a strong impact on containment efforts and the perceived severity of the pandemic, which likely affects the ability and willingness to respond to a survey request. As the US 2020 Decennial Census showed, response rates to a household survey were significantly lower in counties with higher infection rates than in counties with lower infection rates (Bates and Zamadics 2021). COVID-19 related variables are also likely to be informative of changes in firms’ operational activities, which may not only affect the likelihood of participation, but also correlate with the substantive survey variables, including job vacancies, future expectations, or COVID-19 related survey items. Thus, they seem to be suitable candidates to be included in nonresponse adjustment schemes.

## Data and methods

### Data

#### IAB job vacancy survey

The IAB-JVS collects data on a range of topics from job vacancies and worker flows to working hours and recruitment processes. It is designed as a voluntary, nationally-representative, annual repeated cross-sectional survey with a sub-annual panel component (Bossler et al. 2020). In the fourth quarter (October-December) of each year, a new quasi-panel is started with a fresh sample. The full sample consists of about 110,000 establishments drawn from the population of all establishments in Germany with at least one regular employee liable for social security contributions. A stratification by region, industry, and establishment size is applied, resulting in unequal inclusion probabilities. The first panel wave is carried out using a concurrent mixed-mode design with paper questionnaires and an online option. The first panel wave in the fourth quarters of 2017, 2018, and 2019 yielded response rates of about 13 percent and net samples of 14,596, 14,506, and 13,895 observations, respectively.

A subsample of respondents from the first panel wave (i.e. the fourth quarter of each year) are reinterviewed in each of the following three quarters by telephone to update the number of vacancies and key information about working hours. Interviews conducted in the second and third quarters of 2020 included several questions about the impact of the COVID-19 crisis on the establishment. The survey institute set a goal of at least 9,000 completed interviews for each quarterly follow up. Unaware of the upcoming pandemic, a change of telephone studio was implemented in 2020 because the previous telephone studio ceased operations and could no longer carry out the survey. The field protocol and telephone procedures, however, remained the same from previous years and the interviews were monitored for quality assurance. To account for possible differences in the subsampling procedure for the reinterviews, we use weights to balance the fielded samples between the three observation years. (see Sect. 4.2.1 for more information about the weighting approach). This study uses a preliminary dataset of the 2020 IAB-JVS, which was later adjusted to remove two interviewers who were found to have unusually short interviews. The recruitment process itself was not affected by these interviewers (for more information, see Additional file 1: Appendix E.3).

The primary source of telephone numbers and contact persons for the reinterviews is the first panel wave, where respondents are asked for this information at the end of the questionnaire. If the contact information is missing, telephone numbers are sourced using web scraping or information from the Federal Employment Agency. The source of the contact information is likely associated with participation in the reinterviews, as establishments that voluntarily provided their contact information in the first panel wave should have a higher contact rate and willingness to participate than establishments whose contact information had to be sourced from elsewhere. For at least two reasons, providing a contact person in the first panel wave might be positively correlated with the likelihood of participation in the quarterly follow-up interviews. First, establishments that provided their contact information might be more cooperative, and second, the availability of specific contact persons reduces the response burden because the most knowledgeable person does not have to be identified again. In order to account for a possible influence of the phone number source and/or the contact person, we apply two different approaches. First, this information is included in the weighting approach, and second, sensitivity tests are performed based on the subset of establishments that reported this information in the questionnaire.

This study concentrates on the three reinterviews of the 2019 panel conducted in the first, second, and third quarters of 2020 that coincided with the pandemic. To enable comparisons with previous years of the IAB-JVS, we also analyze the corresponding quarterly reinterviews for the panels starting in 2017 and 2018. This year-to-year comparison is based on the assumption that the compositions of the starting net samples are comparable. This assumption holds as evidenced in Additional file 1: Appendix B, which shows there are no substantial differences between these survey years with respect to response rates and respondent composition in the starting samples.

#### The establishment history panel

The Establishment History Panel (BHP) of the Federal Employment Agency (Ganzer et al. 2020) is a longitudinal administrative database, which can be linked to the IAB-JVS using a unique establishment identifier. These data contain rich information on establishments and aggregate employee characteristics and are used as the basis to investigate nonresponse bias in the IAB-JVS. The ${30}^{\mathrm{th}}$ of June each year serves as the reference date for the aggregation of employee characteristics to the establishment level. To analyze nonresponse bias for the three quarterly reinterviews in 2020, the BHP records from 2019 are used as this was the year the panel was initially recruited. This approach has the advantage that nearly all establishments can be included in the analysis and the same administrative information used for all follow-up quarters. Exceptions are establishments that cease to exist between the reference dates of the sample selection (${31}^{\mathrm{th}}$ December of the previous year) and the BHP, which applies to 3.6 percent of all establishments. These establishments are excluded from the nonresponse bias analyses, but are included in the fieldwork and response rate analyses. Table 1 provides an overview of the used variables, including establishment characteristics, employee characteristics, regional information, paradata, and COVID-19 variables. Moreover, it shows which variables are used to answer each research question. Additional file 1: Appendix A describes the used variables in more detail, and Additional file 1: Appendix C presents descriptive statistics. All employee characteristic variables are divided into relatively equal-sized categories based on their distributions.^3^

**Table 1:** Variable and dataset overview

	Biasanalysis (RQ2/4)	Participation models (RQ3)	Weighting schemes (RQ4)			
			Current variables	COVID-19 variables	Admin. variables	All variables
Establishment characteristics (BHP)						
East/West Germany	X	-	-	-	X	X
Federal state	-	X	-	-	-	-
Foundation year	X	X	-	-	X	X
Industry	X	X	X	X	X	X
Establishment founded in sampling year	X	X	-	-	X	X
Number of employees	X	X	X	X	X	X
Employee characteristics (BHP)						
Avg. age of employees	X	-	-	-	X	X
Prop. of female employees	X	-	-	-	X	X
Prop. of fixed-term contracts	X	-	-	-	X	X
Prop. of apprentices	X	-	-	-	X	X
Prop. of full-time contracts	X	-	-	-	X	X
Prop. of part-time contracts	X	-	-	-	X	X
Prop. of German citizens	X	-	-	-	X	X
Prop. of regular contracts	X	-	-	-	X	X
Prop. of marginal contracts	X	-	-	-	X	X
Prop. of high-educated employees	X	-	-	-	X	X
Prop. of mid-educated employees	X	-	-	-	X	X
Prop. of low-educated employees	X	-	-	-	X	X
Prop. of unknown educated employees	X	-	-	-	-	-
Quartile of wage distribution	X	-	-	-	X	X
Regional information						
Inhabitants in municipality	-	X	-	-	-	-
Paradata						
Early vs. late respondent (4th quarter)	-	X	X	X	X	X
Telephone number provided in q’naire	-	X	-	-	-	-
Contact person provided in q’naire	-	X	-	-	-	-
COVID-19 variables						
Avg. COVID-19 incidence	-	X	-	X	-	X
Avg. mobility reduction	-	X	-	X	-	X
Containment regulations	-	X	-	X	-	X
Short-time work	X	X	-	X	-	X

#### COVID-19 data

To analyze establishment survey participation, we analyze four variables that are closely related to the pandemic situation. First, we analyze whether survey participation is associated with regional COVID-19 outbreaks. To do this, we average the official COVID-19 7-day incidence at the administrative district level from the Robert Koch Institute (Robert-Koch-Institute 2021) by quarter and link them to the location of the establishment. Next, we use data from the COVID-19 Mobility Project (Schlosser et al. 2020, 2021) to investigate whether participation is correlated to mobility change at the district level in 2020. Using mobile phone data, the mobility of individuals between cell towers is averaged and compared with the previous year. To analyze survey participation with respect to containment regulations, we exploit an IAB database on containment regulations at the industry level (Bauer and Weber 2021). Finally, administrative data on short-time work benefits at the establishment level are used to assess whether survey participation is correlated with receiving this government subsidy (Statistik der Bundesagentur für Arbeit 2021). The short-time work administrative variable is also used in the nonresponse bias analysis to directly estimate the magnitude of nonresponse bias for the same variable measured in the IAB-JVS.

### Methods

#### Fieldwork outcomes

The first research question (RQ1) compares the fieldwork outcomes in the three quarterly follow-up surveys of 2020 with those of the preceding years 2018 and 2019. Specifically, we compare response rates, cooperation rates, contact rates, and the number of call attempts per completed interview. All rates are computed following the AAPOR definitions of the corresponding rates (American Association for Public Opinion Research 2016) and the corresponding formulas are shown in the Additional file 1: Appendix E.

We define the response rate (RR1) as the proportion of fielded establishments that completed the interview and contact rate (CON1) as the proportion of fielded establishments that were successfully contacted. The cooperation rate (COOP1) is the proportion of contacted establishments that completed the interview. The number of call attempts per completed interview is simply calculated as the ratio of the total number of call attempts and the total number of completed interviews.

To facilitate comparisons between different years and overcome issues of confounding, we use a weighting approach to correct for three possible sources of selection: unequal probabilities of selection, nonresponse in the initial recruitment wave of the 4th quarter, and subsampling of fielded establishments in the corresponding quarter. Design weights are used to adjust for unequal selection probabilities. Nonresponse weights are computed as the inverse of the predicted response propensity in the fourth quarter based on the current IAB-JVS weighting scheme (Brenzel et al. 2016). Similarly, we account for the fact that not all establishments were fielded in the follow-up quarters by estimating fielding propensities using logistic regression and using the inverse of the predicted propensities as the fielding weight. Paradata from the initial panel waves and establishment characteristics (e.g. establishment size, industry) are used as predictors in this estimation. The final adjustment weight, which is used in all analyses, is derived by multiplying the design weight, nonresponse weight, and fielding weight. See Additional file 1: Appendix D for a more detailed description of the weighting scheme. This weighting approach enables us to compute population estimates.

#### Nonresponse bias

The second research question (RQ2) focuses on the analysis of nonresponse bias for each administrative variable listed in Table 1. Nonresponse bias is estimated as the difference between the estimate of interest based on respondents and the corresponding estimate based on the fielded sample:

1 $$\begin{aligned} {\widehat{\text {NR bias}}}_{i} = {\hat{Y}}_{i, r} - {\hat{Y}}_{i, n}\; \end{aligned}$$

where ${\hat{Y}}_{i, r}$ denotes the estimator for the $i^{th}$ statistic of interest based on the respondents and ${\hat{Y}}_{i, n}$ is the estimator based on the fielded sample.

To facilitate comparisons between years, we further generate estimates of absolute nonresponse bias:

2 $$\begin{aligned} {\widehat{\text {Abs. NR bias}_{i}}} = \vert {\widehat{\text {NR bias}_{i}}}\vert \end{aligned}$$

and average absolute nonresponse bias, which is an aggregate nonresponse bias measure calculated across all administrative variables:

3 $$\begin{aligned} {\widehat{\text {Avg. abs. NR bias}}}= \dfrac{\sum _{i=1}^{K} {\widehat{\text {Abs. NR bias}_{i}}}}{K} \end{aligned}$$

where *K* is the total number of statistics for which nonresponse bias is estimated.

Additionally, we report average absolute nonresponse biases separately for two variable groups: establishment characteristics and employee characteristics (see Table 1). Separating these variable groups sheds light on which is most impacted by nonresponse bias and the extent to which responding establishments differ from the full sample on these characteristics. Nonresponse bias on these characteristics is also analyzed as part of the fourth research question (RQ4) evaluating different weighting schemes for nonresponse bias adjustment.

#### Modeling survey participation

To address the third research question (RQ3), we test whether certain types of establishments changed their participation behavior during the COVID-19 pandemic compared to previous years. To do this, we estimate logistic regression models of survey participation for the first, second, and third quarters of 2018, 2019, and 2020, as logistic regression models are commonly used for this purpose (e.g. Struminskaya and Gummer 2022; Blom et al. 2017; Gummer and Struminskaya 2020). The model formulas can be found in the Additional file 1: Appendix G.

In a first step we estimate baseline models using a core set of variables, including industry, establishment size, foundation year, paradata (e.g. the provision of contact person and telephone number in the fourth quarter interview), and a set of control variables, including federal states and number of inhabitants in an establishment’s municipality. Afterwards the coefficients of the separate yearly regressions are tested pairwise if they are statistical significance different using a Wald type significance test based on the assumption that the coefficients of the two separate regressions are uncorrelated due to independent sampling. This step provides evidence whether the participation behavior of certain types of establishments changed in the COVID-19 year relative to the pre-COVID-19 years.

In the second step, we test whether the COVID-19 variables are significant predictors of survey participation. For each quarter of 2020, we estimate three models. First, we include only the four COVID-19 predictor variables in the model. Second, we add establishment characteristics (industry and establishment size, inhabitants in municipality, federal states, foundation year, establishment founded in sampling year), and paradata (early vs. late respondents, contact person provided, telephone number provided) as control variables. Third, we interact short-time work with establishment size and industry. This interaction sheds light on whether the impact of the COVID-19 variables on survey participation differs between large and small establishments or different industries.

All analyses are carried out in Stata 16 (StataCorp 2019) and account for stratification and the aforementioned adjustment weights for unequal inclusion probabilities, nonresponse in the initial recruitment wave, and quarter-specific fielding probabilities.

#### Comparison of weighting schemes for nonresponse adjustment

To address the fourth research question (RQ4), we evaluate the extent to which including additional auxiliary variables (beyond the standard IAB-JVS auxiliary variables) in the nonresponse weighting procedure reduces nonresponse bias in the quarterly follow-ups of the pandemic year (2020) compared to those of 2018 and 2019. Specifically, we fit separate logistic regressions using the following sets of variables for each quarter (see also Table 1):

- *Current weighting variables*
- *Current weighting variables + COVID-19 data (2020 only)*
- *Current weighting variables + administrative data*
- *Current weighting variables + COVID-19 data + administrative data (2020 only)*

The sets containing COVID-19 auxiliary data will only be assessed for year 2020, as these data are not available in previous years. From each regression, response propensities are estimated and inverted to obtain nonresponse adjustment weights. The final adjustment weights used in this analysis are constructed by multiplying the design weight, nonresponse weight from the initial recruitment wave, the quarter-specific fielding weight, and the derived quarter-specific nonresponse weight.

The final adjusted weights are then used to compute estimates of nonresponse bias by comparing the adjusted weighted estimates against the unadjusted (for nonresponse in the quarterly reinterviews) fielded sample estimates. This comparison is done to evaluate how the alternative weighting schemes perform in terms of reducing nonresponse bias in 2020. In addition, we provide model fit statistics to assess the fit of the response propensity estimations. To avoid overfitting, we leave out the variable on which nonresponse bias is estimated from the response propensity estimation. Using this “leave-one-out” approach, different sets of response propensities are computed for each target variable of interest.

## Results

### Fieldwork and participation outcomes

**Fig. 1:**
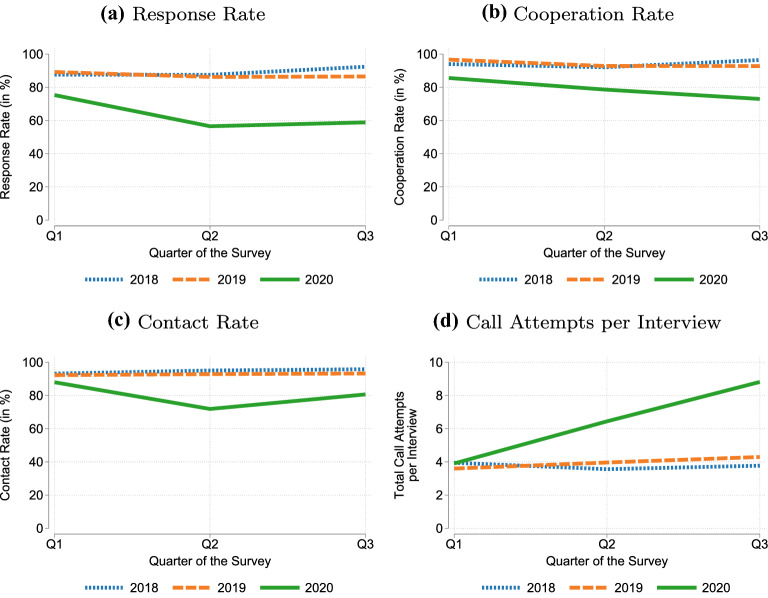
Participation rates and fieldwork effort, 2018-2020 IAB-JVS

First, we examine the response rates of 2020 and the two preceding years as part of the first research question (RQ1). The response rates (Fig. 1a) clearly dropped in 2020. While in 2018 and 2019 the response rates were between 80-90 percent in each quarter, in 2020 the response rate decreased to 75% in quarter 1, 57% in quarter 2, and 59% in quarter 3. This was also reflected in lower numbers of respondents and the fact that the survey was unable to meet its target of 9,000 respondents despite fielding a larger sample compared to previous years (see Additional file 1: Appendix Table E.2). Similarly, the cooperation rate (Fig. 1b) and the contact rate (Fig. 1c) was lower in 2020 compared to previous years. Hence, establishments could not be contacted and refused at a higher rate than in previous years. The response, cooperation, and contact rates in the first quarter of 2020 were more similar to previous years than were the second and third quarters. This pattern may reflect the unfolding of the pandemic, with only about one month (March) of the first quarter affected, while the other two quarters were fully affected by the pandemic.

To assess the intensity of the fieldwork, the average number of call attempts per completed interview is shown (Fig. 1d). In line with previous results, the fieldwork had to be intensified to recruit establishments. In 2018, 2019, and the first quarter of 2020, about four call attempts, on average, were necessary to complete one interview. This increased to more than six in the second quarter and more than eight in the third quarter of 2020. Hence, the COVID-19 pandemic clearly led to more fieldwork effort. Additional file 1: Appendix E shows the corresponding tables and, as a robustness check supporting these results, the same analyses under the condition that respondents provided a telephone number and a contact person in the initial panel wave questionnaire. Based on this robustness check, we can rule out the possibility that the present results are driven by year-to-year differences in the provision of contact information.

Due to the survey’s stratified sampling design and unequal sampling fraction within strata, some establishments in certain establishment size classes and industries have a relatively high likelihood of getting repeatedly sampled in consecutive years of the IAB-JVS. This fact enables us to compare response rates across establishments with and without previous experience with the survey in the two preceding years. As shown in Table 2, establishments that were fielded in either 2018 or 2019, or both, have a higher response rate in 2020 than establishments that weren’t fielded in 2018 and 2019. However, it is evident that the response rates of all groups of establishments – regardless of their experience with the IAB-JVS in 2018 and 2019 – dropped in the second and third quarters of 2020 compared to the first quarter. This pattern wasn’t observed in previous years, where all groups of establishments had comparable response rates in all three quarters. For example, while the response rate of establishments that were fielded in 2018, 2019, and 2020 were in all three quarters of 2019 between 95 and 97 percent, the response rate of this group of establishments dropped in 2020 from 92 (Q1) to 76 (Q2) and 86 (Q3) percent. Similarly, the response rate of establishments that were not fielded in 2018 or 2019 decreased from 75 (Q1) to 56 (Q2) and 58 (Q3) percent in 2020. Hence, the participation rates of establishments with and without previous IAB-JVS experience in 2019 or 2018 were similarly affected in 2020, albeit at different levels.

**Table 2:** Response rates (in %) of establishments fielded in consecutive years of the IAB-JVS

	2018			2019			2020			N (2020)		
	Q1	Q2	Q3	Q1	Q2	Q3	Q1	Q2	Q3	Q1	Q2	Q3
Fielded in 2020 only	-	-	-	-	-	-	75	56	58	8,759	10,880	10,566
Fielded in 2018 & 2020	95	92	91	-	-	-	83	66	73	667	742	713
Fielded in 2019 & 2020	-	-	-	91	90	90	85	68	69	807	984	1,001
Fielded in 2018, 2019 & 2020	98	95	98	97	95	96	92	76	86	431	516	514
Total	88	87	92	89	86	87	75	57	59	10,664	13,122	12,794

Weighted for unequal inclusion probability, nonresponse in the initial recruitment wave and quarter-specific fielding probabilities

### Nonresponse bias

**Fig. 2:**
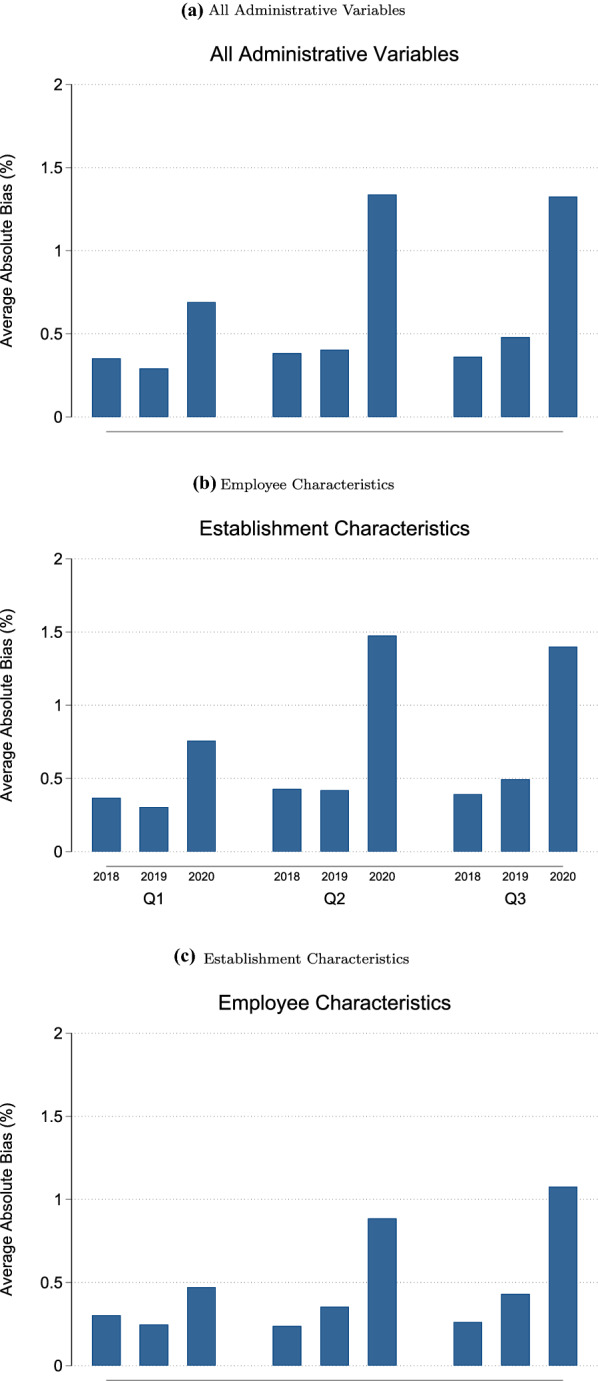
Average absolute nonresponse bias, BHP 2018-2020

Pertinent to research question 2 (RQ2), nonresponse biases in 2020 are compared to 2018 and 2019. Figure 2 plots the average absolute nonresponse bias for all variables (Fig. 2a), and separately for employee characteristics (Fig. 2b) and establishment characteristics (Fig. 2c). Additional file 1: Appendix F contains the corresponding tables of average absolute nonresponse bias estimates and tables of single absolute nonresponse bias estimates.

In each quarter of 2020 the average absolute nonresponse biases were above the corresponding estimates of the preceding two years. The strongest increase was observed in the second and third quarters, which is consistent with the lower response rate noted previously. The average absolute bias reaches 0.68 percentage points in the first quarter, 1.31 percentage points in the second quarter, and 1.33 percentage points in the third quarter. Despite the nearly two-fold increase in the latter quarters, these levels of nonresponse bias are relatively low overall and thus the increased risk of biased substantive analyses should be negligible. Both variable groups, establishment and employee characteristics, show a similar trend and clearly suggest that the average nonresponse bias was higher in 2020 than in the two previous years. Employee characteristics show a higher level of aggregate nonresponse bias than the establishment characteristics.

Taking a closer look at variable-specific nonresponse bias reveals that some variables have larger biases in 2020 compared to previous years. For instance, the proportion of full-time employees (see Additional file 1: Appendix Table F.13) or the proportion of high-educated employees (see Additional file 1: Appendix Table F.16) show bias values up to 5 percentage points in 2020. In the two previous years, the bias estimates for these two variables did not exceed 1.5 percentage points. The number of employees displays a comparably strong increase and the largest bias in 2020 with values up to 3 percentage points compared to a maximum of 0.9 percentage points in the previous years. This is particularly concerning as establishment size is strongly correlated to the number of job vacancies or hirings, which are key variables in the IAB-JVS.

Nonresponse biases for receipt of short-time work benefits are not particularly high in any of the three quarters of 2020 (Additional file 1: Appendix Table F.23). The nonresponse bias estimate is 0.93 percentage points in Q1, 0.21 percentage points in Q2, and 1.26 percentage points in Q3. Hence, the risk that substantive analyses of short-term work will be compromised by nonresponse bias is likely to be low.

### Patterns of survey participation

To address research question 3 (RQ3), we first present the significant pairwise differences of coefficients from separate logistic regressions of survey participation for each quarter of 2018, 2019, and 2020 (see Table 3). Additional file 1: Appendix G shows the detailed results from the logistic regressions, including coefficients, standard errors, and the results of the pairwise significance tests comparing the coefficients of the different survey years.

**Table 3:** Pairwise significance tests of differences between survey participation coefficients by quarter and year, 2018–2020

	Quarter 1			Quarter 2			Quarter 3		
Yearly Comparisons	2018 vs. 2019	2018 vs. 2020	2019 vs. 2020	2018 vs. 2019	2018 vs. 2020	2019 vs. 2020	2018 vs. 2019	2018 vs. 2020	2019 vs. 2020
Industry (Ref.: Agricultural/production)									
Energy/construction/logistic	-	-	-	-	-	-	-	-	-
Retail/hospitality/entertainment	-	-	-	-	-	-	-	-	-
Finance/information/real estate	-	-	-	–/*	–/*	-	-	-	-
Public/educ./health	–/**	–/*	-	-	-	–/*	+/*	-	–/*
Other services	-	-	-	-	-	-	-	-	-
Number of employees (Ref.: 1-9)									
10–19	-	-	-	-	-	-	-	-	-
20–49	-	-	-	-	-	-	-	-	-
50–249	+/*	+/*	-	-	-	-	-	-	-
$\ge$ 250	-	-	-	-	-	-	-	-	-
Foundation year (Ref.: 70s/80s)									
90s	-	-	-	-	-	-	+/*	-	–/*
00s	-	-	-	-	-	-	-	–/*	-
10s	-	-	-	-	-	-	-	-	-
Federal state aggregated (Ref.: Schleswig-Holstein + Hamburg)									
Lower Saxony + Bremen	-	-	-	-	-	-	-	+/*	-
North Rhine-Westphalia	-	-	+/*	-	-	-	-	+/*	-
Hesse	-	-	-	-	-	-	-	-	-
Rhineland-Palastine + Saarland	-	-	-	-	-	-	+/*	+/*	-
Baden-Wuerttemberg	-	-	-	-	-	–/**	-	-	-
Bavaria	-	-	-	-	-	-	-	-	-
Brandenburg + Berlin	-	-	-	-	-	-	-	-	-
Mecklenburg-Vorpommern	-	+/*	-	-	-	-	-	-	-
Saxony	-	-	-	-	-	-	-	-	-
Saxony-Anhalt	-	-	-	-	-	-	-	-	-
Thuringia	-	-	-	-	-	-	-	-	-
Inhabitants in municipality (Ref.: < 15,000)									
15,000–99,999	-	-	-	-	-	-	+/*	+/*	-
$\ge$ 100,000	-	-	-	-	-	-	+/*	-	-
Establishment founded in sampling year	-	-	–/*	-	-	-	-	-	-
Early vs. late respondent (4th quarter)	-	-	-	-	-	-	-	-	-
Contact person provided in q’naire	-	-	-	-	-	-	-	-	-
Telephone number provided in q’naire	-	–/*	–/*	–/*	–/*	-	-	-	-
Number of significant different coefficients	2	4	3	2	2	2	5	5	2

Notes: “+” indicates that difference of coefficients between the most recent year and the base year is positive. Hence, it is more likely than in the previous year.

“–” indicates that difference of coefficients between the most recent year and the base year is negative. Hence, it is less likely than in the previous year.

Significance Levels: * $$p<0.05$$, ** $$p<0.01$$, *** $$p<0.001$$

In the second and third quarters of 2020, the likelihood of participation among establishments working in the industry groups public administration/education/health differs significantly from their counterparts in 2019. They are significantly less likely to participate than the reference industry groups agriculture/production compared to 2019. A possible explanation for the lower likelihood of participation could be that many of these publicly-financed establishments were under special pressure to continue working at full capacity, including hospitals, schools, and local federal employment agencies. Participating in a survey may have been too burdensome for these establishments, resulting in higher nonresponse. Further, the results show that the likelihood of participation among larger establishments (50-249 employees), finance/information/real estate industries, public/education/health industries, and foundation year are statistically significantly different in 2020 compared to some quarters of the previous years. However, differences for these characteristics are also seen between the non-pandemic years (2018/2019). Thus, it is unlikely these differences are driven by the pandemic.

In summary, the comparison of these regression models does not provide indications of a dramatic change in survey participation behavior in 2020 for specific establishment groups. Although some 2020 patterns deviate significantly from previous years, the deviation is not special in any meaningful way.

**Table 4:** Odds-ratios of survey participation for COVID-19 related predictors, by model specification and quarter, 2020

Model	(1)	(2)	(3)	(4)	(5)	(6)	(7)	(8)	(9)
	Q 1 -	Q 1 -	Q 1 -	Q 2 -	Q 2 -	Q 2 -	Q 3 -	Q 3 -	Q 3-
	COVID-19	Core	Interaction	COVID-19	Core	Interaction	COVID-19	Core	Interaction
DV: Survey participation	OR (SE)	OR (SE)	OR (SE)	OR (SE)	OR (SE)	OR (SE)	OR (SE)	OR (SE)	OR (SE)
Short-time work (Ref.: no short-time work)									
Short-time work	0.782	0.771	1.435	1.020	1.000	1.050	1.259*	1.206	1.449*
	(0.121)	(0.122)	(0.380)	(0.093)	(0.094)	(0.159)	(0.138)	(0.138)	(0.260)
Containment-regulations (Ref.: No Containment)									
Containment	0.866	0.874	0.902	0.772	0.715*	0.730	2.149	2.123	2.049
	(0.152)	(0.181)	(0.191)	(0.106)	(0.116)	(0.121)	(1.201)	(1.181)	(1.131)
COVID-19 case incidence (Ref.: 1. quartile)									
2. quartile	1.135	1.127	1.124	0.822	0.807	0.808	1.348*	1.196	1.202
	(0.179)	(0.202)	(0.201)	(0.098)	(0.110)	(0.109)	(0.167)	(0.183)	(0.184)
3. quartile	1.064	0.920	0.912	0.887	0.907	0.910	1.163	1.058	1.065
	(0.161)	(0.166)	(0.166)	(0.116)	(0.133)	(0.133)	(0.141)	(0.177)	(0.178)
4. quartile (lowest incidence)	1.172	0.883	0.879	1.048	0.954	0.955	1.159	1.045	1.048
	(0.173)	(0.172)	(0.172)	(0.124)	(0.146)	(0.146)	(0.169)	(0.200)	(0.200)
Mobility reduction (Ref.: 1. quartile (most reduction))									
2. quartile	1.108	1.164	1.162	0.914	0.980	0.989	0.906	0.952	0.947
	(0.166)	(0.183)	(0.183)	(0.112)	(0.151)	(0.153)	(0.113)	(0.145)	(0.144)
3. quartile	1.291	1.400*	1.389*	1.024	1.191	1.194	1.075	1.102	1.091
	(0.186)	(0.216)	(0.216)	(0.121)	(0.204)	(0.204)	(0.143)	(0.189)	(0.187)
4. quartile (least reduction/increase)	1.240	1.248	1.240	0.882	1.138	1.149	0.973	1.109	1.103
	(0.183)	(0.213)	(0.212)	(0.116)	(0.218)	(0.220)	(0.130)	(0.196)	(0.195)
Industry (Ref.: Agriculture/production)									
Energy/construction/logistic		0.973	1.039		0.742**	0.810		0.718**	0.757*
		(0.131)	(0.148)		(0.077)	(0.100)		(0.077)	(0.091)
Retail/hospitality/entertainment		0.967	1.023		0.944	0.947		0.775*	0.816
		(0.176)	(0.200)		(0.135)	(0.171)		(0.099)	(0.121)
Finance/information/real estate		0.858	0.909		0.887	0.962		1.120	1.236
		(0.126)	(0.138)		(0.104)	(0.132)		(0.138)	(0.168)
Public/educ./health		0.847	0.872		0.767*	0.791		0.766*	0.778
		(0.140)	(0.154)		(0.090)	(0.111)		(0.092)	(0.102)
Other services		0.959	1.089		1.064	1.235		1.024	1.002
		(0.160)	(0.205)		(0.137)	(0.203)		(0.136)	(0.149)
Number of employees (Ref.:1-9)									
10–19		1.277	1.306		1.389**	1.192		1.139	1.076
		(0.170)	(0.187)		(0.151)	(0.155)		(0.126)	(0.131)
20–49		1.173	1.280*		1.420***	1.320**		1.432***	1.516***
		(0.127)	(0.151)		(0.118)	(0.131)		(0.123)	(0.143)
50–249		1.515**	1.587**		1.510***	1.374**		1.708***	1.758***
		(0.204)	(0.238)		(0.157)	(0.168)		(0.178)	(0.212)
$\ge$250		1.394*	1.406*		1.260*	1.196		1.172	1.176
		(0.217)	(0.228)		(0.145)	(0.163)		(0.138)	(0.164)
Industry $\times$ Short-time work									
Energy/construction/logistic $\times$ short-time work			0.525			0.771			0.749
			(0.212)			(0.176)			(0.194)
Retail/hospitality/entertainment $\times$ short-time work			0.571			0.934			0.769
			(0.198)			(0.223)			(0.206)
Finance/information/real estate $\times$ short-time work			0.620			0.735			0.516*
			(0.314)			(0.187)			(0.160)
Public/educ./health $\times$ short-time work			0.715			0.982			1.114
			(0.344)			(0.256)			(0.425)
Other services $\times$ short-time work			0.435*			0.687			1.256
			(0.165)			(0.175)			(0.393)
Number of employees $\times$ *short-time work*									
10–19 $\times$ short-time work			0.868			1.556*			1.430
			(0.337)			(0.344)			(0.400)
20–49 $\times$ short-time work			0.626			1.239			0.795
			(0.184)			(0.218)			(0.179)
50–249 $\times$ short-time work			0.731			1.308			0.876
			(0.239)			(0.279)			(0.209)
$\ge$250 $\times$ short-time work			0.815			1.114			0.913
			(0.352)			(0.241)			(0.218)
Constant	2.554***	1.328	1.258	1.496**	0.855	0.815	1.195	0.943	0.907
	(0.365)	(0.400)	(0.380)	(0.199)	(0.249)	(0.239)	(0.170)	(0.285)	(0.275)
Observations	10664	10664	10664	13122	13122	13122	12794	12794	12794
Pseudo $R^{2}$	0.005	0.029	0.030	0.003	0.027	0.028	0.004	0.025	0.028
AIC	2209738	2155792	2153035	2710562	2647523	2642495	2679979	2622335	2616723
BIC	2209804	2156069	2153377	2710629	2647807	2642847	2680046	2622618	2617073

Exponentiated coefficients; Standard errors in parentheses

* $$p<0.05$$, ** $$p<0.01$$, *** $$p<0.001$$

Core Variables in Models (2), (3), (5), (6), (8), (9): Federal state, foundation year, inhabitants in municipality, establishment founded in sampling year, early vs. late respondent (4th quarter), contact person provided in q’naire, telephone number provided in q’naire

Weighted for unequal inclusion probabilities, nonresponse in the initial recruitment wave, and quarter-specific fielding probabilities

Table 4 shows the results of separate logistic regressions of survey participation for each quarter of 2020 with a focus on analyzing the predictive power of the COVID-19 characteristics. We start by discussing the base models (Models 1,4,7) and then discuss how they change after adding the core set of variables (Models 2,5,8) and interaction terms (Models 3,6,9). The containment regulation variable is not statistically significant in any of the three quarters (Models 1,4,7). In the third quarter (Model 7), establishments with short-time work are more likely to participate than establishments without short-time work. Moreover, establishments in regions with higher incidences are more likely to participate than establishments in regions with lower incidences. When the core variables are added to the baseline model of the first quarter (Model 2), the coefficient of the mobility reduction variable becomes statistically significant. Hence, establishments in regions with less mobility reduction have a higher likelihood of participation than establishments in regions with the strongest mobility reduction in the first quarter. Similarly, the coefficient for containment regulations becomes statistically significant in the second quarter (Model 5), suggesting that establishments subject to containment regulations are less likely to participate than establishments not subject to containment regulations. After adding the core variables to the third quarter model (Model 8), the effects of short-time work and COVID-19 incidence lose their statistical significance. The interaction model of the first quarter (Model 3) shows that establishments in industry group Other Services and receiving short-time work benefits have a lower likelihood of response. While the containment regulations variable is no longer statistically significant in the second quarter interaction model (Model 6), the interaction term of short-time work and establishments with 10 to 19 employees becomes statistically significant. This means that establishments with 10 to 19 employees and receiving short-time work have a particularly high likelihood of participation. In the third quarter (Model 9), the interaction term of establishments in industry group Finance/Information/Real Estate and short-time work is significantly negative, suggesting that these establishments have a particularly lower likelihood of participation compared to the other establishments.

In summary, some COVID-19 related variables (e.g. short-time work, COVID-19 case incidence in the third quarter) are significant predictors of survey participation in 2020, though the effects are inconsistent across the three observed quarters. In addition, some of the variables lose their statistical significance after controlling for basic establishment characteristics. Moreover, the COVID-19 variables (Models 1,4,7) improve the model fit only marginally (Mc-Fadden’s pseudo-R$^{2}$; Q1: 0.005, Q2: 0.003, Q3: 0.004).

### Nonresponse weighting adjustment

**Fig. 3:**
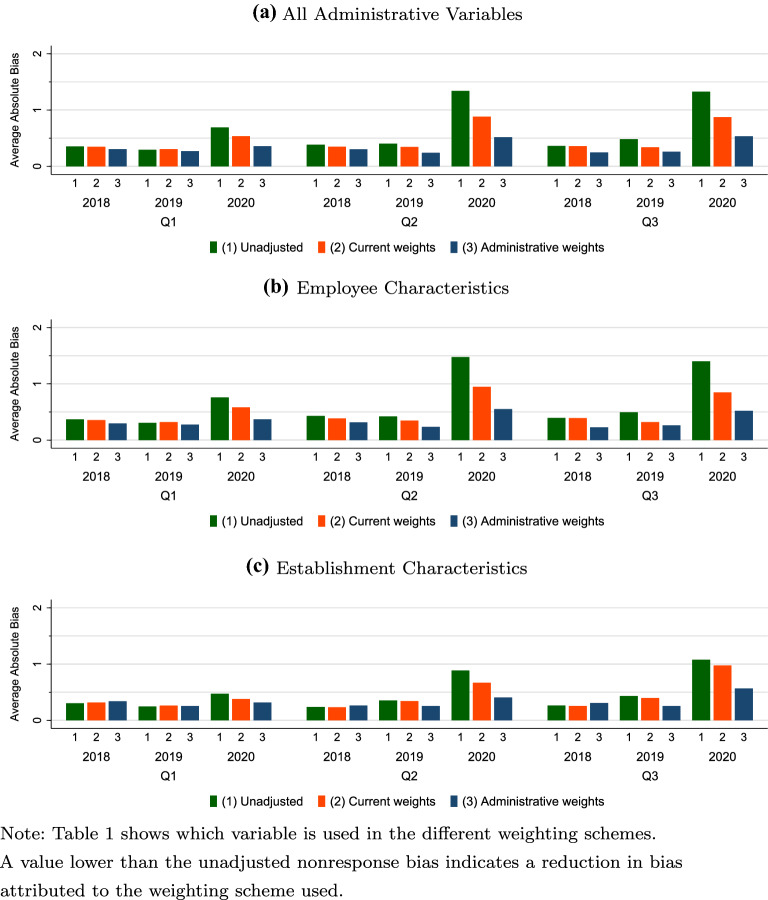
Average absolute nonresponse bias by weighting scheme, BHP 2018-2020. Table 1 shows which variable is used in the different weighting schemes. A value lower than the unadjusted nonresponse bias indicates a reduction in bias attributed to the weighting scheme used.

As shown in Sect 4.2, nonresponse biases were larger in 2020 compared to 2019 and 2018, on average. This raises the question (RQ4) whether this increased bias can be reduced with the current weighting scheme or an alternative weighting scheme based on additional auxiliary variables. To answer this research question, we compare in a first step the average absolute nonresponse bias estimates under the current IAB-JVS weighting scheme with an alternative weighting scheme that incorporates additional administrative variables for the first, second, and third quarters of 2020 and the corresponding quarters of the preceding years. Figure 3 shows the average absolute nonresponse bias for the target administrative variables after applying the different weighting schemes. The bias results are shown for all target administrative variables (Fig. 3a), and separately for the employee characteristics (Fig. 3b) and establishment characteristics (Fig. 3c). In Additional file 1: Appendix H, the corresponding tables for average absolute biases and adjusted single bias estimates are provided. The figure represents the unadjusted nonresponse bias (bar 1), the adjusted nonresponse bias under the current weighting scheme (bar 2), and the adjusted nonresponse bias under the alternative weighting scheme which incorporates an extended set of administrative variables (bar 3).

First, we evaluate the weighting schemes on the average absolute nonresponse bias across all administrative variables (Fig. 3a). The results reveal that the current adjustment procedure substantially reduces the nonresponse bias in all three quarters of 2020, but not to the same level as that of the preceding years. Adding more administrative variables to the weighting scheme leads to a further substantial reduction of nonresponse bias and more than halves the nonresponse bias compared to the unadjusted case and further closes the gap with the observed nonresponse bias of the preceding years. All separate quarter-specific analyses show the same pattern and support the conclusion that adding more administrative weighting variables seems to improve upon the current adjustment procedure for addressing nonresponse bias during the pandemic year. Although the results show that the weighting adjustments are effective in reducing nonresponse bias in 2020, the remaining nonresponse bias is still slightly higher compared to the preceding years, and especially in the second and third quarters. The same conclusions hold when looking at the employee characteristics (Fig. 3b) and establishment characteristics (Fig. 3c) separately.

Looking at the single bias estimates (see Additional file 1: Appendix H.3), the extended administrative data weighting scheme performs very well in reducing nonresponse bias for the number of employees, proportion of full-time contracts, and proportion of fixed-term contracts in 2020. For the number of employees, the extended weighted scheme reduces the bias estimates in 2020 to a similar level as in 2018 and 2019. The model fit statistics, provided in the Additional file 1: Appendix H.4, also support the conclusion that the additional administrative data improves the response propensity estimation used in the weighting scheme. By looking at the response propensity models (see Additional file 1: Appendix H.5), we identify administrative variables that are especially useful. Aside from establishment size, the following employee characteristics employees seem to be especially fruitful: proportion of high-educated employees and proportion of female.

In the second step, we examine whether adding the COVID-19 variables to the current weighting scheme improves nonresponse bias reduction in 2020. Additional file 1: Appendix Figure H.1 shows the average absolute bias for all target administrative variables (Figure H.1a, and separately for employee characteristics (Figure H.1b) and establishment characteristics for 2020 (Figure H.1c). Although the COVID-19 variables were previously shown to be somewhat predictive of survey participation (Table 4), the results of the weighting analysis provide no evidence that incorporating COVID-19 variables into the weighting adjustment reduces nonresponse bias in any of the three quarters of 2020. This is also true for both variable groups: employee characteristics and establishment characteristics. Hence, the extended administrative variables (see Figs. 3a–c) seem to be the only additional auxiliary variables that are effective in reducing nonresponse bias relative to the current IAB-JVS weighting scheme.

## Discussion

This article investigated survey participation, nonresponse bias, and the effectiveness of alternative nonresponse bias adjustments during the COVID-19 pandemic in a nationally-representative establishment survey, i.e. the IAB-Job Vacancy Survey (IAB-JVS). As the comparison with preceding years showed, survey participation in 2020 was negatively affected by the pandemic, resulting in lower response rates, higher refusal rates, lower contact rates, and more intensive fieldwork efforts resulting in more calls per completed interview. In addition, we observed greater nonresponse bias than in preceding years based on available administrative data. However, despite the larger nonresponse bias during the pandemic year, the level of nonresponse bias was still rather low, suggesting that it does not pose a major threat to the IAB-JVS.

The survey participation models showed that the response patterns of establishments did not substantially differ between the COVID-19 year and the pre-COVID-19 years. However, one interesting difference was that in 2020 establishments working in the public administration, education, and health sectors were less likely to participate compared to previous years, likely because these sectors were operating under especially difficult circumstances during the pandemic. Establishments receiving short-time work benefits were more likely to participate in the third quarter than establishments that did not receive short-time work. This finding is inline with the results of Kagerl et al. (2022), who also found that establishments receiving short-time work benefits have a higher response rate than establishments that did not receive short-time work benefits.

The final analysis of this article evaluated alternative sets of weighting variables for nonresponse bias adjustment. First, the evaluation showed that the current set of weighting variables reduced nonresponse bias in 2020, but not to the same level that was observed prior to the pandemic. A further bias reduction was achieved by adding extensive administrative variables capturing establishment and employee characteristics. Adding COVID-19 related variables was ineffective in reducing nonresponse bias. These results are in line with Küfner et al. (2022) and Rothbaum et al. (2021) who showed that administrative auxiliary variables improve nonresponse adjustment procedures compared to standard weighting schemes. Nonetheless, it is notable that the even the best weighting scheme was still unable to reduce aggregate nonresponse bias in 2020 to a comparable level observed in previous years. This suggests that 2020 was indeed a unique year for establishment survey participation with more differential nonresponse than has been seen in years past.

This study has several implications for survey practice. We showed that the COVID-19 pandemic reduced response rates and likely increased nonresponse bias in the voluntary IAB-Job Vacancy Survey. Survey organizations and researchers should consider whether their surveys were similarly affected. In doing so, it is important to assess which types of establishments were most likely affected by pandemic-related nonresponse and their potential impacts on substantive research results. Another practical import of this study is that COVID-19 related variables, though somewhat predictive of survey participation, are rather ineffective as auxiliary variables in nonresponse bias adjustments. In contrast, survey statisticians should consider utilizing more administrative data in their adjustment procedures. Examples of administrative variables that we found to be particularly useful were establishment size and industry, and characteristics of the workforce, including the proportions of high-educated employees and female employees. Adjustment weights based on these administrative variables could lead to a further reduction in nonresponse bias. The present study could serve as a blueprint for survey practitioners to investigate the impact of COVID-19 or other powerful events on survey participation and nonresponse bias. In addition, a strategy to re-evaluate the auxiliary variables used in nonresponse weighting schemes is suggested.

We note some study limitations. First, we analyzed an establishment survey in Germany, where the lockdown measures were relatively mild compared to other countries (e.g. Spain, France, Italy). Countries with stricter containment measures may have affected survey participation even more strongly. Moreover, the first wave of the IAB-JVS was already underway before the COVID-19 pandemic hit the world. Hence, the first survey contact was made without any influence of the COVID-19 pandemic. The observed effects on survey participation might have been even more prominent if establishments were first approached during the pandemic (see for example Kagerl et al. 2022).

## Conclusion

In conclusion, this study showed that the 2020 COVID-19 pandemic year negatively affected survey participation, fieldwork effort, and led to greater nonresponse bias compared to previous years. Further, we showed that the participation behavior of certain types of establishments changed during the pandemic, and that COVID-19 related variables are only marginally associated with the likelihood of participation. Including extensive administrative data in nonresponse weighting schemes appears to reduce the pandemic-related effects of differential nonresponse, but does not completely resolve the issue of increased nonresponse bias in 2020. We advise survey organizations to be aware of such issues in their own surveys and evaluate different adjustment approaches and sources of auxiliary data to minimize the biasing effects of pandemic-related nonresponse in their establishment surveys.

## Supplementary Information

**Additional file 1**.

## Data Availability

The IAB-JVS and BHP administrative datasets are available from the Research Data Centre (RDC) of the Federal Employment Agency in Germany. Restrictions apply to the availability of these data, which are not publicly available. For more information on data access, see https://fdz.iab.de/en.aspx.

## Footnotes

1 The importance of analyzing nonresponse during the pandemic was also emphasized by Gummer (2020) in his commentary about the impacts of the COVID-19 pandemic on labor market surveys at the German Institute for Employment Research (Sakshaug et al. 2020): “Consequently, revisiting the IAB surveys and investigating how COVID-19 affected survey outcomes in more detail will be a relevant task for the future.”

2 see Kagerl et al. (2022) for more information about short-time work and issues measuring it in establishment surveys.

3 The IAB-JVS and BHP administrative datasets are available from the Research Data Centre (RDC) of the Federal Employment Agency in Germany. Restrictions apply to the availability of these data, which are not publicly available. For more information on data access, see https://fdz.iab.de/en.aspx.

## References

[CR1] Altig, D., Barrero, J. M., Bloom, N., Davis, S. J., Meyer, B. H., Mihaylov, E., Parker, N.: Survey of business uncertainty - methodology (2020). https://www.atlantafed.org/-/media/documents/research/surveys/business-uncertainty/survey-of-business-uncertainty-methodology.pdf”. Accessed 03 May 2021

[CR2] American Association for Public Opinion Research. Standard Definitions: Final Dispositions of Case Codes and Outcome Rates for Surveys. AAPOR, 9th edition (2016)

[CR3] Bates N, Zamadics J (2021). Covid-19 infection rates and propensity to self-respond in the 2020 us decennial census. Surv. Pract..

[CR4] Bauer A, Weber E (2021). Covid-19: how much unemployment was caused by the shutdown in Germany?. Appl. Econ. Lett..

[CR5] Blom AG, Herzing JM, Cornesse C, Sakshaug JW, Krieger U, Bossert D (2017). Does the recruitment of offline households increase the sample representativeness of probability-based online panels? evidence from the german internet panel. Soc. Sci. Comput. Rev..

[CR6] Bossler M, Gürtzgen N, Kubis A, Küfner B, Lochner B (2020). The IAB Job Vacancy Survey: design and research potential. J. Labour Market Res..

[CR7] Brenzel H, Czepek J, Kiesl H, Kriechel B, Kubis A, Moczall A, Rebien M, Röttger C, Szameitat J, Warning A (2016). Revision of the IAB-Job Vacancy Survey: Backgrounds, Methods and Results.

[CR8] Brinca, P., Duarte, J. B., Faria-e-Castro, M.: Is the covid-19 pandemic a supply or a demand shock? Econ. Synop. (31)(2020)

[CR9] Davis, W. R., Pihama, N.: Survey response as organisational behaviour: an analysis of the annual enterprise survey, 2003-2007 (2009). Paper presented at New Zealand Association of Economists Conference, Wellington

[CR10] Donthu N, Gustafsson A (2020). Effects of covid-19 on business and research. J. Bus. Res..

[CR11] Earp M, Toth D, Phipps P, Oslund C (2018). Assessing nonresponse in a longitudinal establishment survey using regression trees. J. Off. Stat..

[CR12] Ganzer, A., Schmidtlein, L., Stegmaier, J., Wolter, S.: Establishment History Panel 1975-2019. FDZ-Datenreport 16/2020, Research Data Centre of the German Federal Employment Agency, Nürnberg (2020)

[CR13] Gummer T (2020). Commentary on impacts of the covid-19 pandemic on labor market surveys at the german institute for employment research. Surv. Res. Methods.

[CR14] Gummer, T., Struminskaya, B.: Early and late participation during the field period: Response timing in a mixed-mode probability-based panel survey. Soc. Methods Res. 1–24 (2020)

[CR15] Gürtzgen, N., Kubis, A.: Stellenbesetzungen in der Corona-Krise: Mehr Arbeitslose pro Offene Stelle, weniger Besetzungsschwierigkeiten. 15, IAB-Kurzbericht (2021)

[CR16] Hecht V, Litzel N, Schäffler J (2019). Unit nonresponse at the firm level: a cross-border analysis using the IAB-ReLOC data. J. Labour Market Res..

[CR17] HMRC. Large Business Methodology Review. Her Majesty’s Revenue and Customs Research Report 98, Ipsos MORI (2010)

[CR18] Janik F (2011). Unit Non-response in Establishments Surveyed for the First Time in the IAB Establishment Panel.

[CR19] Janik F, Kohaut S (2012). Why don’t they answer? Unit non-response in the IAB establishment panel. Qual. Quant..

[CR20] Kagerl C, Schierholz M, Fitzenberger B (2022). Later one knows better: the over-reporting of short-time work in firm surveys. J. Labour Market Res..

[CR21] König C, Sakshaug JW, Stegmaier J, Kohaut S (2021). Trends in establishment survey nonresponse rates and nonresponse bias: evidence from the 2001–2017 IAB establishment panel. J. Off. Stat..

[CR22] Kožuh, L. Š.: Data collection in the light of covid-19 (2021) Paper presented at European Establishment Statistics Workshop

[CR23] Küfner, B., Sakshaug, J. W., Zins, S.: Analyzing establishment survey nonresponse using administrative data and machine learning. J Royal Stat. Soc.: Series A (2022, forthcoming)

[CR24] Lineback, J.F., Thompson, K.J.: Conducting nonresponse bias analysis for business surveys, In: Proceedings of the American Statistical Association, Government Statistics Section pp. 317–331 (2010)

[CR25] Little RJ, Vartivarian S (2005). Does weighting for nonresponse increase the variance of survey means?. Surv. Methodol..

[CR26] McKenzie, M.: Response rates AES. Private Communication (2021)

[CR27] Moreira, A., Lima, B., Poças, J., Magalhães, J., Cruz, P., Gil, S., Rodrigues, S.: The use of electronic invoice data in covid time (2021). Paper presented at European Establishment Statistics Workshop

[CR28] Phipps, S., Jones, C.: Factors affecting response to the occupational employment statistics survey. In: Proceedings of the 2007 Federal Committee on Statistical Methodology Research Conference (2007)

[CR29] Phipps P, Toth D (2012). Analyzing establishment nonresponse using an interpretable regression tree model with linked administrative data. Ann. Appl. Stat..

[CR30] Robert-Koch-Institute. Covid-19 (2021). https://www.rki.de/EN/Content/infections/epidemiology/outbreaks/COVID-19/COVID19.html. Accessed 18 May 2021.

[CR31] Rothbaum, J., Eggleston, J., Bee, A., Klee, M., Mendez-Smith, B.: Addressing nonresponse bias in the american community survey during the pandemic using administrative data. American Community Survey Research and Evaluation Report Memorandum Series ACS21-RER-05, U.S. Census Bureau, Washington (2021)

[CR32] Sakshaug JW, Beste J, Coban M, Fendel T, Haas G-C, Hülle S, Kosyakova Y, König C, Kreuter F, Küfner B (2020). Impacts of the covid-19 pandemic on labor market surveys at the German institute for employment research. Surv. Res. Methods.

[CR33] Schlosser, F., Maier, B. F., Hinrichs, D., Zachariae, A., Brockmann, D.: Covid-19 lockdown induces structural changes in mobility networks–implication for mitigating disease dynamics. arXiv preprint arXiv:2007.01583 (2020) 10.1073/pnas.2012326117 PMC7776901 33273120

[CR34] Schlosser, F., Maier, B., Jack, O., Hinrichs, D., Zachariae, A., Brockmann, D.: Covid-19 mobility germany (2021) 10.1073/pnas.2012326117 PMC7776901 33273120

[CR35] Seiler C (2014). The determinants of unit non-response in the Ifo Business Survey. AStA Wirtschafts-und Sozialstatistisches Archiv.

[CR36] Snijkers, G.: The effect of response measures in business surveys (2018). Paper presented at Conference of European Statisticians, Geneva

[CR37] Snijkers G, Haraldsen G, Jones J, Willimack D (2013). Designing and Conducting Business Surveys.

[CR38] Stapleton, M., Sun, H., Hoverman, V., Levin, K., Bedford, B., Miller, S., Kraft, B.: Comparison of respondents and non-respondents to a covid-19 impacts survey of businesses (2021). Paper presented at American Association for Public Opinion Research

[CR39] StataCorp. Stata Statistical Software: Release 16. College Station. College Station, TX (2019)

[CR40] Statistik der Bundesagentur für Arbeit. Tabellen, Realisierte Kurzarbeit, Daten mit einer Wartezeit von bis zu 5 Monaten (ohne Hochrechnung) (2021)

[CR41] Struminskaya B, Gummer T (2022). Risk of nonresponse bias and the length of the field period in a mixed-mode general population panel. J. Surv. Stat. Methodol..

[CR42] Thompson KJ, Oliver BE (2012). Response rates in business surveys: going beyond the usual performance measure. J. Off. Stat..

[CR43] Tomaskovic-Devey D, Leiter J, Thompson S (1995). Item nonresponse in organizational surveys. Sociol. Methodol..

[CR44] U.S. Bureau of Labor Statistics. Household and establishment survey response rates (2021). https://www.bls.gov/osmr/response-rates/. Accessed 29 Apr 2021.

[CR45] Valliant R, Dever JA, Kreuter F (2013). Practical Tools for Designing and Weighting Survey Samples.

[CR46] Weir, D.: HRS update. Unpublished Workshop Presentation (2020)

