# Supplementary material for: Establishment survey participation during the COVID-19 pandemic
Source: J Labour Mark Res. 2022 Nov 13;56(1):18. doi: 10.1186/s12651-022-00321-8 (PMC9660198; doi:10.1186/s12651-022-00321-8)
Supplement: Supplementary file 1 — Additional file 1. [file 12651_2022_321_MOESM1_ESM.pdf]

Online Appendix

Appendix A: Variables

A.1 Establishment characteristics (BHP)

Federal State

The German federal states are captured by the BHP variable *ao_bula*. In order to build fairly equal sized categories, smaller states are combined with larger states. For instance, Bremen is combined with Lower Saxony. This variable is used in the survey participation models (RQ3).

East/West Germany

Nonresponse bias (RQ2, RQ4) with respect to region is measured with a dummy variable differing between West and East Germany. This variable is based on the federal states and the division of the federal states was aligned with the division of Germany before 1990.

Foundation Year

The foundation year of the establishment is grouped into four categories. The first category includes establishments founded in 1970s and 1980s, the second category includes establishments founded in the 1990s, the third category includes establishments founded in the 2000s, and the fourth category consists of establishments founded in the 2010s. The BHP variable *grd_dat* forms the basis for the categorized variable that is used in the survey participation models (RQ3) and nonresponse bias measures (RQ2, RQ4).

Industry

The economic activity of an establishment is captured by the BHP variable *w08_3* with a 3-digit code. For the purpose of the survey participation models, a distinction is made between six different groups of industries: "Agriculture/Production", "Energy/Construction/Logistic", "Retail/Hospitality/Entertainment", "Finance/Information/Real Estate", "Public/Education/Health", and "Other Services". Nonresponse bias is measured on a different categorized variable with three categories: "Agriculture/Production", "Service" and "Public Administration, Education, Health and Arts".

Establishment founded in sampling year

The BHP data indicates whether an establishment was likely to be founded in the sampling year. This can occur if the establishment has no employees subject to social security in the year prior to the sampling year. However, in some cases this conclusion is not true. For instance, establishments could have changed their administrative employment identifier for administrative reasons or due to merging with another establishment.

Number of Employees

Establishment size is measured by the number of employees working in an establishment, independent of their working hours. This information is captured by the BHP variable *az_ges*. To investigate the impact of the number of employees on survey participation, we use a variable with six categories: Less than 9 employees, 10-19 employees, 20-49 employees, 50-249 employees, and more than 250 employees. In order to analyze nonresponse bias (RQ2, RQ4), we use only four categories: less then 10 employees, between 10 and 19 employees, between 20 and 49 employees, and at least 50 employees.

A.2 Employee Characteristics (BHP)

Avg. Age of Employees

The BHP variable (*alter_mw*) is categorized into four equal sized groups to construct a variable for the average age of employees: 0-38.99 years; 39.00-34.49 years; 43.50-47.99 years; and $\geq$48.00 years.

Proportion of Female Employees

The number of employees is captured in the BHP variable *az_f*. This information is converted into a categorical variable that relates the number of female employees to the total number of employees and divides them into four equally sized categories: 0-18.75 percent of female employees, 18.76 - 44.43 percent of female employees, 44.44 - 71.41 percent of female employees, and more than 71.42 percent of female employees.

Proportion of Fixed-Term Contracts

The BHP also captures the number of employees with fixed-term contracts (*az_bf*). As for the proportion of female employees, the number of employees with fixed-term contracts are divided by the number of employees and then split into into three categories: 0 percent, 0.01-15.99 percent, and more than 16.00 percent of employees with a fixed-term contract.

Proportion of Apprentices

The proportion of apprentices captures the ratio of apprentices (*az_azubi*) divided by the total number of employees. To assess nonresponse bias, we use a dummy variable that differs between establishments without apprentices and establishments with at least one apprentice.

Proportion of Full-Time Contracts

Another aggregate employee variable captured by the BHP is the number of employees with full-time contracts (*az_vz*). We divide this information by the number of all employees to get comparable statistics across all establishments. Afterwards we build four equally-sized groups for the nonresponse bias analysis: Less than 37.14 percent of employees with full-time contracts, between 37.15 and 66.66 percent, between 66.67 and 86.04 percent, and establishments with more than 86.05 percent of employees with full-time contracts.

Proportion of Part-Time Contracts

As with the proportion of full-time employees, a variable is also created for employees with part-time contracts. Hence, we divide the number of part-time employees (*az_tz*) by the number of all employees and allocate these values into three equally sized-group: Zero employees with part-time contracts, between 0.01 and 19.99 percent, and more than 20.00 percent of employees with part-time contracts.

Proportion of German Citizens

The BHP variable *az_d* covers the number of German citizens among the employees. Again, we relate this information to the total number of employees and create a dummy variable for establishments with 100 percent German employees and with at least one non-German employee.

Proportion of Regular Contracts

Another aggregate employee characteristic is the proportion of employees with regular contracts as defined in the BHP (Ganzer et al., 2020). The proportion of employees with regular contracts relates the number of employees with regular contracts to the total number of employees and is categorized into four groups: Less than 71.24 percent of employees with regular contracts, between 71.25 and 87.49 percent, between 87.50 and 96.90 percent, and more than 96.91 percent of employees with regular contracts.

Proportion of Marginal Contracts

Based on the BHP variable *az_gf*, the proportion of marginal employees in relation to the total number of employees is generated. This variable classifies establishments into the following categories: no marginal employees, below 0.01 - 14.99 percent, and more than 15.00 percent of marginal employees.

Proportion of High-Educated Employees

The BHP distinguishes three education levels of employees: high-educated, middle-educated, and low-educated. The proportion of employees with the corresponding education level is calculated for each establishment, which are used for the bias analysis (RO2, RO4). The share of high-educated employees is grouped into establishments with 0 percent of high-educated employees, between 0.01 - 14.99 percent, and more than 15.00 percent of high-educated employees.

Proportion of Mid-Educated Employees

The categorized variable of middle-educated employees consists of the following groups: 0-55.25 percent of middle-educated employees, 55.26-74.99 percent, 75.00-88.88 percent, and more than 88.89 percent of middle-educated employees.

Proportion of Low-Educated Employees

Establishments are divided into the following categories in terms of low-educated employees: 0 percent of low-educated employees, between 0.01 - 11.99 percent, and more than 12.00 percent of low-educated employees.

Proportion of Unknown-Educated Employees

Next to the three aforementioned education levels, one can also compute the number of employees with missing information on their educational level. In line with the previously described variables, the proportion is computed and divided into – in this case – two categories: 0 percent of employees with an unknown education level, and 0.01 percent or more with an unknown education level.

Quartile of Wage Distribution

The BHP offers different wage information. For this study the median wage per establishment (*te_imp_med*) is used, where censored wages above the limit for social security contributions are imputed. The median is less affected by outliers and the use of the imputation procedure provides a better picture for the establishment as a whole. Unfortunately, the wage information is only available for establishments with at least one full-time employee. All establishments without any full-time employees are grouped into a missing category. To have comparable results between the years and be robust to inflation, we created indicators that assign each establishment each year to a quarter of the wage distribution of the population. This variable is used for the bias analysis and the adjustment models.

A.3 Regional Information

Inhabitants in Municipality

Previous research has illustrated that the structure of the area the establishment is located, mostly the number of inhabitants, is associated with survey participation. To take this factor into account, the municipality of the location is linked to the official number of inhabitants in that municipality provided by Destatis (2021). Afterwards we compute a variable with three different categories: Less than 15,000 inhabitants, between 15,001 and 99,999 inhabitants, and more than 100,000 inhabitants.

A.4 Paradata

Early vs. Late Respondent (4th Quarter)

As described in Section 3.1.1, that analysis consists of establishments that have already participated in the initial IAB-JVS recruitment wave, i.e. the fourth quarter. As one important paradata information that may affect the likelihood of participation in the three quarterly reinterviews, we distinguish between establishments that answered early in the fourth quarter and those that answered late in the fourth quarter. To distinguish both groups, we split the set of respondents at the median of the response date distribution into early and late respondents. A small fraction of establishments did not provide an answer on the question when they answered the questionnaire. These cases are counted as late respondents as they are more likely to be reluctant respondents (Loosveldt and Billiet, 2002). This dummy variable is then used in the survey participation models (RQ3) and in the response propensity estimations (RQ4).

Telephone Number Provided in Questionnaire

As also discussed in Section 3.1.1, the source for the telephone number is likely to be correlated with the likelihood of participation in the quarterly reinterviews. There are three possible sources. The first and most important source for telephone numbers is the questionnaire of the initial panel wave. If the telephone number is missing in that questionnaire, telephone numbers are supplemented by web scraping or information from the Federal Employment Agency. To consider the source of the telephone number, we use a dummy variable for each establishment, indicating whether the establishment provided a telephone number in the questionnaire of the initial panel wave. This dummy variable is used for sensitivity checks of research question 1 and in the survey participation models (RQ3).

Contact Person Provided in Questionnaire

Similar to the telephone number provision, respondents of the first panel wave were asked to provide the name of a contact person. This name is used in the recruitment of the person for the reinterviews. We expect that a voluntarily provided contact name is associated with a higher likelihood of participation in the reinterviews. Hence, we also use this information in the sensitivity checks for the participation outcomes (RQ1) and the survey participation models (RQ3). This information is also captured in a dummy variable, distinguishing between establishments that provided the name of a contact person and those that did not.

A.5 COVID-19 variables

Avg. COVID-19 Incidence

COVID-19 cases are defined as persons who tested positive for COVID-19. To capture the intensity of the current outbreak across different administrative districts, the daily number of COVID-19 cases are averaged across the last seven days and standardized among 100,000 inhabitants for each administrative district independently. In this study, we average them further into a quarter-specific average of COVID-19 case incidences and build four equally-sized groups based on their distribution. Using the district information of the establishment, COVID-19 case incidence is merged to the establishment level data. The source for these statistics is the Robert-Koch-Institute.

Avg. Mobility Reduction

The COVID-19 pandemic led to a massive decline in mobility due in part to increasing remote working. A project run by Humboldt-University Berlin measures the mobility of individuals using mobile phone data at the district level. To this end, they capture the movement of mobile phones between cell towers. The mobility reduction is then captured by comparing the values of 2019 with those of 2020. We average these mobility reduction statistics by quarter and link them to the establishment data with the help of the administrative district. Afterwards we categorize this variable into four equally-sized groups based on its distribution.

Containment Regulations

An IAB database captures containment regulations at the industry level. To gather quarterly data, this database is restructured into a dummy variable whether the industry was affected by containment regulations in this quarter or not. Based on a 5-digit industry code, this containment variable is merged to the establishment level data.

Short-Time Work

Short-time work is a subsidy for establishments negatively affected by the pandemic. The subsidy is granted by the Federal Employment Agency. The short-time work variable is recorded at the establishment level and indicates whether or not an establishment received short-time work benefits in a quarter. It is therefore structured as a dummy variable.

Appendix B: Comparisons of Respondents of the Initial Panel Wave

Year-to-year comparisons are based on the assumption that the initial recruitment waves between 2017 and 2019 are comparable (see B.1). The size of the full sample was the same in all three years. The response rate differs only by 0.7 percentage points between 2017 and 2019. Based on a design-based F-Test, differences between the three observation years in terms of administrative variables are found only for the proportion of employees with unknown education, the proportion of apprentices, industry, and foundation year. The latter differs by definition as the foundation year is classified based on the foundation decade. Hence, the 2019 survey has more establishments from 2010s than the 2017 survey. The proportion of employees with unknown education and the proportion of apprentices differs mostly between 2017 and 2018/2019. The 2020 net sample consists of slightly more establishments in the industry group "Public Administration/ Education/ Health/Arts" than in the service sector compared to previous years. However, the absolute difference is small, with values up to a maximum of 1.5 percentage points. All in all, the initial recruitment wave for the 2020 follow-up interviews is comparable to the two preceding years.

Table B.1 Recruitment Panel Wave by Year, 2017 - 2019

	2017	2018	2019
Full Sample (N)	14,596	14,506	13,895
Net Sample (n)	109,900	109,889	109,898
Response Rate (in %)	13.3	13.2	12.6

Unweighted Response Rate

Appendix C: Descriptive Statistics

Table C.1 Descriptive Statistics Q4-Respondents - Avg. Age of Employees, BHP

	2017			2018			2019		
Avg. Age of Employees	unwgt. Obs.	wgt. Obs.	wgt. Prop.	unwgt. Obs.	wgt. Obs.	wgt. Prop.	unwgt. Obs.	wgt. Obs.	wgt. Prop.
0.00-38.99	3,054	589,511	29.57 %	2,989	591,692	29.69 %	2,814	548,298	27.61 %
39.00-43.49	3,516	392,919	19.71 %	3,396	385,242	19.33 %	3,245	388,569	19.56 %
43.50-47.99	4,046	384,897	19.31 %	3,937	385,011	19.32 %	3,814	394,741	19.87 %
≥48.00	3,726	626,385	31.42 %	3,912	630,940	31.66 %	3,771	654,614	32.96 %
Design Based F-Test:	0.683								

Table C.2 Descriptive Statistics Q4-Respondents - Prop. of Female Employees, BHP

	2017			2018			2019		
Prop. of Female Employees	unwgt. Obs.	wgt. Obs.	wgt. Prop.	unwgt. Obs.	wgt. Obs.	wgt. Prop.	unwgt. Obs.	wgt. Obs.	wgt. Prop.
0.00 - 18.18	3,757	454,632	22.80 %	3,595	443,265	22.24 %	3,504	461,571	23.24 %
18.19 - 41.93	3,661	334,979	16.80 %	3,812	369,511	18.54 %	3,632	351,148	17.68 %
41.94 - 68.38	3,393	390,482	19.59 %	3,419	392,517	19.70 %	3,148	377,306	19.00 %
>68.39	3,531	813,620	40.81 %	3,408	787,592	39.52 %	3,360	796,198	40.09 %
Design Based F-Test:	0.653								

Table C.3 Descriptive Statistics Q4-Respondents - Prop. of Fixed-Term Contracts, BHP

	2017			2018			2019		
Prop. of Fixed-Term Contracts	unwgt. Obs.	wgt. Obs.	wgt. Prop.	unwgt. Obs.	wgt. Obs.	wgt. Prop.	unwgt. Obs.	wgt. Obs.	wgt. Prop.
0.00	5,178	1,283,350	64.37 %	5,147	1,256,076	63.03 %	5,192	1,267,225	63.80 %
0.01 - 15.99	4,345	258,288	12.96 %	4,333	271,289	13.61 %	4,010	261,149	13.15 %
≥16.00	4,819	452,074	22.67 %	4,754	465,520	23.36 %	4,442	457,849	23.05 %
Design Based F-Test:	0.804								

Table C.4 Descriptive Statistics Q4-Respondents - Prop. of Full-Time Contracts, BHP

	2017			2018			2019		
Prop. of Full-Time Contracts	unwgt. Obs.	wgt. Obs.	wgt. Prop.	unwgt. Obs.	wgt. Obs.	wgt. Prop.	unwgt. Obs.	wgt. Obs.	wgt. Prop.
0.00 - 34.40	3,318	883,196	44.30 %	3,280	847,245	42.51 %	3,269	879,069	44.26 %
34.41 - 65.35	3,578	449,536	22.55 %	3,771	502,028	25.19 %	3,634	454,416	22.88 %
65.36 - 85.15	3,835	310,844	15.59 %	3,817	294,886	14.80 %	3,699	308,235	15.52 %
>85.16	3,611	350,136	17.56 %	3,366	348,727	17.50 %	3,042	344,503	17.34 %
Design Based F-Test:	0.311								

Table C.5 Descriptive Statistics Q4-Respondents - Prop. of Part-Time Contracts, BHP

	2017			2018			2019		
Prop. of Part-Time Contracts	unwgt. Obs.	wgt. Obs.	wgt. Prop.	unwgt. Obs.	wgt. Obs.	wgt. Prop.	unwgt. Obs.	wgt. Obs.	wgt. Prop.
0.00	2,896	590,653	29.63 %	2,863	585,558	29.38 %	2,743	593,769	29.89 %
0.01 - 19.99	5,703	369,490	18.53 %	5,594	369,227	18.53 %	5,315	366,238	18.44 %
≥20	5,743	1,033,569	51.84 %	5,777	1,038,100	52.09 %	5,586	1,026,215	51.67 %
Design Based F-Test:	0.994								

Table C.6 Descriptive Statistics Q4-Respondents - Prop. of German Citizens, BHP

	2017			2018			2019		
Prop. of German Citizens	unwgt. Obs.	wgt. Obs.	wgt. Prop.	unwgt. Obs.	wgt. Obs.	wgt. Prop.	unwgt. Obs.	wgt. Obs.	wgt. Prop.
100.00	7,320	1,352,698	67.85 %	6,960	1,309,430	65.71 %	6,591	1,314,714	66.19 %
0.00-99.99	7,022	641,014	32.15 %	7,274	683,455	34.29 %	7,053	671,508	33.81 %
Design Based F-Test:	0.169								

Table C.7 Descriptive Statistics Q4-Respondents - Prop. of Apprentices, BHP

	2017			2018			2019		
Prop. of Apprentices	unwgt. Obs.	wgt. Obs.	wgt. Prop.	unwgt. Obs.	wgt. Obs.	wgt. Prop.	unwgt. Obs.	wgt. Obs.	wgt. Prop.
0.00	9,166	1,622,152	81.36 %	9,088	1,568,200	78.69 %	8,810	1,586,915	79.90 %
0.01-100	5,176	371,559	18.64 %	5,146	424,685	21.31 %	4,834	399,307	20.10 %
Design Based F-Test:	0.016								

Table C.8 Descriptive Statistics Q4-Respondents - Prop. of High-Educated Employees, BHP

	2017			2018			2019		
Prop. of High-Educated Employees	unwgt. Obs.	wgt. Obs.	wgt. Prop.	unwgt. Obs.	wgt. Obs.	wgt. Prop.	unwgt. Obs.	wgt. Obs.	wgt. Prop.
0.00	4,185	1,091,557	54.75 %	4,249	1,075,956	53.99 %	4,282	1,065,870	53.66 %
0.01 - 14.99	5,049	298,954	14.99 %	4,913	332,511	16.68 %	4,706	319,634	16.09 %
≥15.00	5,108	603,200	30.26 %	5,072	584,418	29.33 %	4,656	600,718	30.24 %
Design Based F-Test:	0.369								

Table C.9 Descriptive Statistics Q4-Respondents - Prop. of Mid-Educated Employees, BHP

	2017			2018			2019		
Prop. of Mid-Educated Employees	unwgt. Obs.	wgt. Obs.	wgt. Prop.	unwgt. Obs.	wgt. Obs.	wgt. Prop.	unwgt. Obs.	wgt. Obs.	wgt. Prop.
0.00 - 50.45	2,969	501,976	25.18 %	3,075	545,038	27.35 %	3,001	551,511	27.77 %
50.46 - 72.79	3,419	383,749	19.25 %	3,513	371,888	18.66 %	3,394	375,869	18.92 %
72.80 - 86.66	3,811	358,716	17.99 %	3,838	367,181	18.42 %	3,658	355,703	17.91 %
>86.67	4,143	749,271	37.58 %	3,808	708,778	35.57 %	3,591	703,139	35.40 %
Design Based F-Test:	0.380								

Table C.10 Descriptive Statistics Q4-Respondents - Prop. of Low-Educated Employees, BHP

	2017			2018			2019		
Prop. of Low-Educated Employees	unwgt. Obs.	wgt. Obs.	wgt. Prop.	unwgt. Obs.	wgt. Obs.	wgt. Prop.	unwgt. Obs.	wgt. Obs.	wgt. Prop.
0.00	5,440	1,166,947	58.53 %	5,213	1,108,628	55.63 %	5,017	1,150,088	57.90 %
0.01 - 11.99	5,004	254,988	12.79 %	5,010	263,750	13.23 %	4,622	272,345	13.71 %
≥12.00	3,898	571,777	28.68 %	4,011	620,507	31.14 %	4,005	563,789	28.38 %
Design Based F-Test:	0.061								

Table C.11 Descriptive Statistics Q4-Respondents - Prop. of Unknown-Educated Employees, BHP

	2017			2018			2019		
Prop. of Unknown-Educated Employees	unwgt. Obs.	wgt. Obs.	wgt. Prop.	unwgt. Obs.	wgt. Obs.	wgt. Prop.	unwgt. Obs.	wgt. Obs.	wgt. Prop.
0.00	9,524	1,547,498	77.62 %	9,029	1,485,707	74.55 %	8,442	1,467,992	73.91 %
0.01-100.00	4,818	446,213	22.38 %	5,205	507,178	25.45 %	5,202	518,230	26.09 %
Design Based F-Test:	0.001								

Table C.12 Descriptive Statistics Q4-Respondents - Prop. of Marginal Contracts, BHP

	2017			2018			2019		
Prop. of Marginal Contracts	unwgt. Obs.	wgt. Obs.	wgt. Prop.	unwgt. Obs.	wgt. Obs.	wgt. Prop.	unwgt. Obs.	wgt. Obs.	wgt. Prop.
0.00	4,576	779,953	39.12 %	4,504	784,365	39.36 %	4,318	781,539	39.35 %
0.00-14.99	5,121	253,833	12.73 %	4,890	257,528	12.92 %	4,554	257,491	12.96 %
≥15	4,645	959,926	48.15 %	4,840	950,992	47.72 %	4,772	947,192	47.69 %
Design Based F-Test:	0.987								

Table C.13 Descriptive Statistics Q4-Respondents - Prop. of Regular Contracts, BHP

	2017			2018			2019		
Prop. of Regular Contracts	unwgt. Obs.	wgt. Obs.	wgt. Prop.	unwgt. Obs.	wgt. Obs.	wgt. Prop.	unwgt. Obs.	wgt. Obs.	wgt. Prop.
0.00 - 70.73	3,337	822,642	41.26 %	3,543	814,204	40.86 %	3,562	827,395	41.66 %
70.74 - 87.57	3,989	394,577	19.79 %	3,946	388,196	19.48 %	3,858	373,809	18.82 %
87.58 - 96.93	3,679	140,811	7.06 %	3,503	141,202	7.09 %	3,163	147,581	7.43 %
>96.94	3,337	635,682	31.88 %	3,242	649,284	32.58 %	3,061	637,437	32.09 %
Design Based F-Test:	0.895								

Table C.14 Descriptive Statistics Q4-Respondents - Wage Distribution, BHP

	2017			2018			2019		
Wage Distribution	unwgt. Obs.	wgt. Obs.	wgt. Prop.	unwgt. Obs.	wgt. Obs.	wgt. Prop.	unwgt. Obs.	wgt. Obs.	wgt. Prop.
First Quartile	2,036	370,711	18.59 %	1,826	377,217	18.93 %	1,818	359,610	18.11 %
Second Quartile	2,920	374,503	18.78 %	2,997	391,046	19.62 %	2,854	384,401	19.35 %
Third Quartile	3,396	389,500	19.54 %	3,416	377,871	18.96 %	3,452	385,586	19.41 %
Fourth Quartile	4,817	371,243	18.62 %	4,898	378,851	19.01 %	4,342	375,000	18.88 %
Missings	1,173	487,755	24.46 %	1,097	467,901	23.48 %	1,178	481,625	24.25 %
Design Based F-Test:	0.981								

Table C.15 Descriptive Statistics Q4-Respondents - Federal States aggregated, BHP

	2017			2018			2019		
Federal States aggregated	unwgt. Obs.	wgt. Obs.	wgt. Prop.	unwgt. Obs.	wgt. Obs.	wgt. Prop.	unwgt. Obs.	wgt. Obs.	wgt. Prop.
Schleswig-Holstein + Hamburg	634	123,274	6.18 %	703	128,753	6.46 %	690	116,015	5.84 %
Lower Saxony + Bremen	1,164	217,039	10.89 %	1,378	238,792	11.98 %	1,305	214,453	10.80 %
North Rhine-Westphalia	1,987	364,143	18.26 %	2,076	341,979	17.16 %	2,110	353,916	17.82 %
Hesse	819	148,829	7.46 %	849	145,266	7.29 %	812	126,203	6.35 %
Rhineland-Palatinate + Saarland	570	100,886	5.06 %	674	107,690	5.40 %	631	110,131	5.54 %
Baden-Wuerttemberg	1,426	274,584	13.77 %	1,645	255,115	12.80 %	1,599	270,659	13.63 %
Bavaria	2,098	350,784	17.59 %	2,244	372,588	18.70 %	2,362	386,225	19.45 %
Brandenburg + Berlin	1,789	134,706	6.76 %	1,524	140,247	7.04 %	1,347	142,688	7.18 %
Mecklenburg-Vorpommern	643	44,979	2.26 %	498	42,799	2.15 %	453	53,306	2.68 %
Saxony	1,497	117,889	5.91 %	1,191	101,832	5.11 %	1,126	107,471	5.41 %
Saxony-Anhalt	788	54,575	2.74 %	671	63,778	3.20 %	537	48,199	2.43 %
Thuringia	927	62,022	3.11 %	781	54,048	2.71 %	672	56,957	2.87 %
Design Based F-Test:	0.569								

Table C.16 Descriptive Statistics Q4-Respondents - Industry, BHP

	2017			2018			2019		
Industry	unwgt. Obs.	wgt. Obs.	wgt. Prop.	unwgt. Obs.	wgt. Obs.	wgt. Prop.	unwgt. Obs.	wgt. Obs.	wgt. Prop.
Agric./Production	5,675	459,540	23.05 %	5,737	457,174	22.94 %	5,563	459,115	23.11 %
Service	4,260	959,073	48.10 %	4,083	944,318	47.38 %	3,963	930,988	46.87 %
Public/Educ./Health/Arts	4,407	575,100	28.85 %	4,414	591,393	29.68 %	4,118	596,119	30.01 %
Design Based F-Test:	0.000								

Table C.17 Descriptive Statistics Q4-Respondents - Foundation Year, BHP

	2017			2018			2019		
Foundation Year	unwgt. Obs.	wgt. Obs.	wgt. Prop.	unwgt. Obs.	wgt. Obs.	wgt. Prop.	unwgt. Obs.	wgt. Obs.	wgt. Prop.
70s/80s	3,883	476,553	23.90 %	4,085	422,666	21.21 %	3,847	375,508	18.91 %
90s	4,544	467,738	23.46 %	4,056	480,468	24.11 %	3,689	458,392	23.08 %
00s	3,401	571,901	28.69 %	3,303	510,497	25.62 %	3,184	533,586	26.86 %
10s	2,514	477,519	23.95 %	2,790	579,254	29.07 %	2,924	618,736	31.15 %
Design Based F-Test:	0.000								

Table C.18 Descriptive Statistics Q4-Respondents - Number of Employees, BHP

	2017			2018			2019		
Number of Employees	unwgt. Obs.	wgt. Obs.	wgt. Prop.	unwgt. Obs.	wgt. Obs.	wgt. Prop.	unwgt. Obs.	wgt. Obs.	wgt. Prop.
1-9	3,804	1,390,475	69.74 %	3,776	1,375,093	69.00 %	3,850	1,379,384	69.45 %
10-19	3,614	297,032	14.90 %	3,930	309,204	15.52 %	3,873	296,071	14.91 %
20-49	3,502	188,631	9.46 %	3,417	189,523	9.51 %	3,319	190,313	9.58 %
$\geq$50	3,422	117,574	5.90 %	3,111	119,065	5.97 %	2,602	120,455	6.06 %
Design Based F-Test:	0.788								

Appendix D: Weighting Scheme

To account for selectivity in the initial panel wave of the cohort study, we apply a weighting scheme using design weights and nonresponse weights. These nonresponse weights are the inverse of the estimated response propensities that are predicted by logistic regressions using region, industry, establishment size, wage of employees, age of employees, and proportion of marginal employees as auxiliary variables. By multiplying design weights and adjustment weights we derive the adjustment weights. In addition, we account for potential selectivity in the fielding process in the follow-up interviews by applying fielding weights. Analogous to response propensities, these are propensity weights from a logistic regression of an establishment being fielded in a particular quarter on the following covariates: industry, region, establishment size, foundation year, response timing (dummy), inhabitants in the city, telephone number provided in the initial panel wave, and contact person provided in the first wave. These fielding weights are multiplied with the adjustment weights of the initial panel wave.

Appendix E: Fieldwork

E.1 Disposition Codes

The fieldwork codes for response, refusal, and noncontacts are generated based on the disposition codes generated in the telephone recruitment process. Table E.1 displays the corresponding outcomes. More detailed disposition codes have been used since the second quarter of 2020.

Table E.1 Disposition Codes

Q1/2018-Q1/2020				Q2/2020-Q3/2020			
**Disposition Code**	**Response**	**Refusal**	**Noncontact**	**Disposition Code**	**Response**	**Refusal**	**Noncontact**
Interview	X	-	-	Interview	X	-	-
Refuses to answer the questions because a similar IAB survey is to be answered in the same time period	-	X	-	Refuses to answer the questions because a similar IAB survey is to be answered in the same time period	-	X	-
Refuses to answer the questions (Other Reasons)	-	X	-	Refuses to answer the questions (Other Reasons)	-	X	-
Establishment no longer exists (business discontinued, insolvency, closure, etc.)	-	-	X	Establishment no longer exists (business discontinued, insolvency, closure, etc.)	-	-	X
Not available during the field phase (after at least 3 attempts to get in touch)	-	-	X	Not available during the field phase (after at least 3 attempts to get in touch)	-	-	X
Number unobtainable, cannot be reached at this phone number	-	-	X	Number unobtainable, cannot be reached at this phone number	-	-	X
Phone number cannot be ascertained	-	-	X	Phone number cannot be ascertained	-	-	X
-	-	-	-	Refuses to answer the questions (COVID-19)	-	X	-
-	-	-	-	No one answers the phone/answering machine	-	-	X
-	-	-	-	Telephone line busy	-	-	X
-	-	-	-	Establishment reached, but person sought or person able to provide information not available for discussion	-	X	-
-	-	-	-	Person sought or person able to provide information is busy at the moment / appointment made	-	X	-

Table E.2 Fieldwork Outcomes by Year and Quarter, 2018 - 2020

	2018			2019			2020		
	Q1	Q2	Q3	Q1	Q2	Q3	Q1	Q2	Q3
Number of Respondents	8,984	8,988	8,989	8,987	8,985	8,986	8,543	8,289	8,546
Number of Refusals	503	495	255	276	490	463	1,183	1,853	2,346
Number of Contacts	9,487	9,483	9,244	9,263	9,475	9,449	9,726	10,142	10,892
Number of Non-Fielded	4,606	4,675	5,039	4,599	4,418	4,532	3,044	550	886
Full Sample	14,596	14,596	14,596	14,506	14,506	14,506	13,895	13,895	13,895
Response Rate (in %)	87.6	87.5	92.4	89.2	86.2	86.5	75.3	56.5	58.9
Cooperation Rate (in %)	94.0	92.1	96.4	96.7	92.8	92.8	85.6	78.6	73.0
Contact Rate (in %)	93.2	95.0	95.8	92.3	92.9	93.3	88.0	71.9	80.6
Fielding Rate (in %)	64.5	64.9	60.4	65.4	67.5	64.9	75.2	94.8	91.7

Rates are weighted for unequal inclusion probability, nonresponse in the initial recruitment wave and quarter-specific fielding probabilities.

E.2 Formulas

The response rate (RR1) is defined as the proportion of fielded establishments that completed the interview and is calculated as:

$$\text{Response Rate} = \frac{I}{(I + R + NC)} \tag{4}$$

where I denotes the number of interviews, R denotes the number of refusals, and NC denotes the number of noncontacts.

The contact rate (CON1) is defined as the proportion of fielded establishments that were successfully contacted. This is expressed as:

$$\text{Contact Rate} = \frac{(I + R)}{(I + R + NC)} \tag{5}$$

The cooperation rate (COOP1) is defined as the proportion of contacted establishments that completed the interview. It is calculated as:

$$\text{Cooperation Rate} = \frac{I}{(I + R)} \tag{6}$$

The number of call attempts per completed interview is simply calculated as the ratio of the total number of call attempts and the total number of completed interviews:

$$\text{No. of Call Attempts per Completed Interview} = \frac{\text{No. Call Attempts}}{I} \tag{7}$$

E.3 Recruitment Process

The recruitment process was unlikely affected by the two interviewers, who were found to have conducted unusually short interviews, for the following reasons. First, the interviewers had no influence on the dialing process. The dialing software decided which establishments to call, not the interviewer. Second, the analysis of the ratio of call attempts to interviews (see Table E.3) showed no evidence that Interviewer 1 and Interviewer 10 were in any way remarkable with regard to the recruitment process. Neither of these interviewers had the highest or lowest ratio of call attempts to interviews. Third, there was no evidence from the interview recordings that either interviewer systematically influenced the recruitment process. Hence, we have no reason to believe that the recruitment process was manipulated by these interviewers.

Table E.3 Call Attempts per Interview by Interviewer, IAB-JVS 2020

Interviewer	Q1	Q2	Q3
1	5.79	5.58	6.58
2	9.13	7.29	10.08
3	9.19	7.53	10.38
4	-	9.75	7.43
6	-	-	8.92
7	17.90	5.92	7.56
10	7.66	-	-
11	6.94	-	-
12	6.00	-	-
13	5.03	-	-
14	10.05	3.97	4.71
16	-	11.75	-

E.4 Sensitivity Checks

In order to account for possible selection effects or varying fieldwork managements, the fieldwork analyses is rerun based on participating establishments that provided contact information in the fourth quarter questionnaire. Hence, no additional research for telephone numbers was carried out for these cases. Figure E.1 summarizes the response rate, cooperation rate, contact rate, and the number of call attempts per interview. The results follow closely the pattern of the unconditional analyses. This supports the conclusion that both participation outcomes and fieldwork effort were negatively affected by the COVID-19 crises.

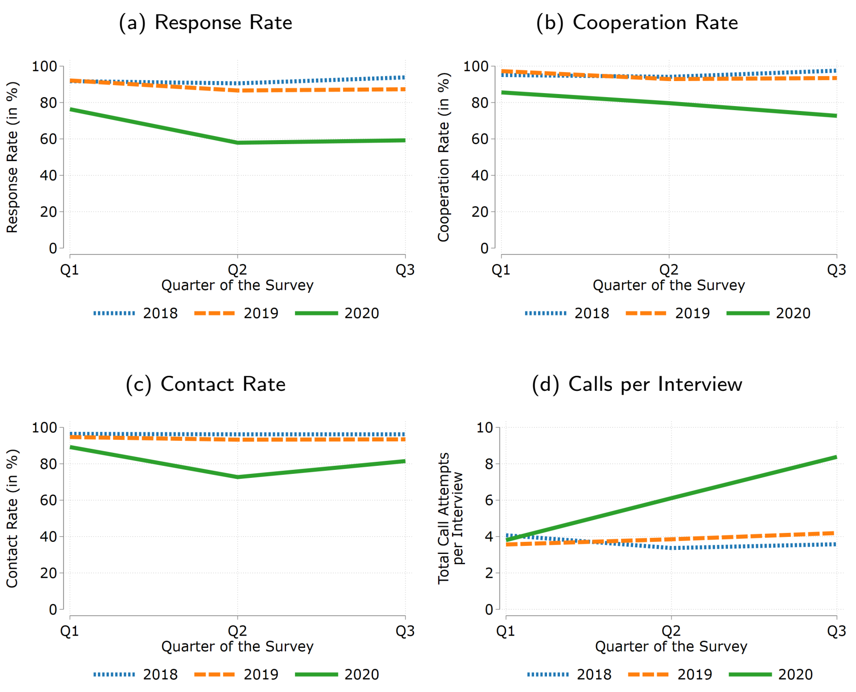

Figure E.1 Participation Rates Conditional on Contact Information in the Questionnaire, 2018-2020 IAB-JVS

Appendix F: Nonresponse Bias

F.1 Average Absolute Bias

Table F.1 Average Absolute Bias by Set of Explanatory Variables, All Administrative Variables BHP 2018-2020

	Q1	Q2	Q3
2018	0.352	0.384	0.362
2019	0.291	0.403	0.480
2020	0.690	1.338	1.325

Table F.2 Average Absolute Bias by Set of Explanatory Variables, Employee Characteristics BHP 2018-2020

	Q1	Q2	Q3
2018	0.367	0.428	0.392
2019	0.305	0.418	0.494
2020	0.757	1.475	1.400

Table F.3 Average Absolute Bias by Set of Explanatory Variables, Establishment Characteristics BHP 2018-2020

	Q1	Q2	Q3
2018	0.303	0.239	0.262
2019	0.248	0.354	0.432
2020	0.472	0.885	1.076

F.2 Single Absolute Biases

Table F.4 Bias Estimates of Foundation Year by Year and Quarter, BHP 2018-2020

Foundation Year	2018			2019			2020		
	Q1	Q2	Q3	Q1	Q2	Q3	Q1	Q2	Q3
70s/80s	0.058	0.093	0.007	0.102	0.039	0.077	0.338	0.990	2.153
90s	0.634	0.034	0.739	0.297	0.671	0.544	0.258	0.013	0.342
00s	0.186	0.573	1.152	0.554	0.205	0.205	0.506	0.523	0.839
10s	0.506	0.700	0.420	0.155	0.427	0.261	0.586	1.526	2.651

Table F.5 Bias Estimates of East/West Germany by Year and Quarter, BHP 2018-2020

East/West Germany	2018			2019			2020		
	Q1	Q2	Q3	Q1	Q2	Q3	Q1	Q2	Q3
East Germany (Ref.: West Germany)	0.047	0.070	0.379	0.470	0.146	0.238	0.010	0.890	1.460

Table F.6 Bias Estimates of Industry by Year and Quarter, BHP 2018-2020

Industry	2018			2019			2020		
	Q1	Q2	Q3	Q1	Q2	Q3	Q1	Q2	Q3
Agric./Production	0.246	0.001	0.063	0.040	0.358	0.202	0.892	0.614	0.090
Service	1.037	0.226	0.004	0.265	0.935	1.176	0.799	0.393	0.165
Public/Educ./Health/Arts	0.791	0.227	0.060	0.305	0.577	0.975	0.093	0.221	0.255

Table F.7 Bias Estimates of Establishment Founded in Sampling Year by Year and Quarter, BHP 2018-2020

Establishment Founded in Sampling Year	2018			2019			2020		
	Q1	Q2	Q3	Q1	Q2	Q3	Q1	Q2	Q3
Founded Before Sampling Year (Ref.: Founded in Sampling Year)	0.110	0.163	0.145	0.223	0.234	0.065	0.039	0.019	0.382

Table F.8 Bias Estimates of Number of Employees by Year and Quarter, BHP 2018-2020

Number of Employees	2018			2019			2020		
	Q1	Q2	Q3	Q1	Q2	Q3	Q1	Q2	Q3
1-9	0.164	0.507	0.098	0.289	0.504	0.937	1.306	3.160	2.828
10-19	0.136	0.087	0.122	0.184	0.006	0.240	0.589	1.358	0.425
20-49	0.002	0.228	0.138	0.114	0.207	0.409	0.235	1.000	1.189
$\geq$50	0.026	0.192	0.082	0.220	0.291	0.288	0.482	0.802	1.214

Table F.9 Bias Estimates of Avg. Age of Employees by Year and Quarter, BHP 2018-2020

Avg. Age of Employees	2018			2019			2020		
	Q1	Q2	Q3	Q1	Q2	Q3	Q1	Q2	Q3
0.00-38.99	0.349	0.280	0.250	0.254	0.056	0.548	0.346	0.661	0.619
39.00-43.49	0.125	0.404	0.143	0.220	0.535	0.589	0.162	0.118	0.071
43.50-47.99	0.163	0.300	0.641	0.466	0.303	0.784	0.778	0.269	0.399
≥48.00	0.387	0.984	0.749	0.432	0.782	0.824	0.270	0.511	0.149

Table F.10 Bias Estimates of Prop. of Apprentices by Year and Quarter, BHP 2018-2020

Prop. of Apprentices	2018			2019			2020		
	Q1	Q2	Q3	Q1	Q2	Q3	Q1	Q2	Q3
0.00 (Ref.: 0.01-100)	0.056	0.521	0.105	0.006	0.765	0.550	0.776	1.740	2.033

Table F.11 Bias Estimates of Prop. of Female Employees by Year and Quarter, BHP 2018-2020

Prop. of Female Employees	2018			2019			2020		
	Q1	Q2	Q3	Q1	Q2	Q3	Q1	Q2	Q3
0.00 - 18.18	0.205	0.454	0.296	0.393	0.396	0.172	0.150	0.766	0.204
18.19 - 41.93	0.405	0.163	0.096	0.216	0.126	0.092	0.918	2.134	1.871
41.94 - 68.38	0.717	0.945	0.207	0.300	0.393	0.093	0.435	1.020	1.106
>68.39	1.327	0.328	0.185	0.477	0.129	0.171	1.203	3.919	3.181

Table F.12 Bias Estimates of Prop. of Fixed-Term Contracts by Year and Quarter, BHP 2018-2020

Prop. of Fixed-Term Contracts	2018			2019			2020		
	Q1	Q2	Q3	Q1	Q2	Q3	Q1	Q2	Q3
0.00	0.697	1.037	0.216	0.120	0.997	1.036	0.369	2.836	2.369
0.01 - 15.99	0.193	0.539	0.051	0.046	0.443	0.127	0.792	1.525	1.793
≥16.00	0.504	0.499	0.165	0.166	0.554	0.908	0.423	1.311	0.576

Table F.13 Bias Estimates of Prop. of Full-Time Contracts by Year and Quarter, BHP 2018-2020

Prop. of Full-Time Contracts	2018			2019			2020		
	Q1	Q2	Q3	Q1	Q2	Q3	Q1	Q2	Q3
0.00 - 34.40	0.039	0.404	0.147	0.248	0.272	0.363	1.694	4.410	3.835
34.41 - 65.35	0.172	0.391	0.790	0.131	0.806	0.520	0.712	2.092	1.430
65.36 - 85.15	0.017	0.250	0.072	0.134	0.045	0.256	0.395	2.035	1.990
>85.16	0.194	0.238	0.571	0.513	0.489	0.412	0.588	0.283	0.415

Table F.14 Bias Estimates of Prop. of Part-Time Contracts by Year and Quarter, BHP 2018-2020

Prop. of Part-Time Contracts	2018			2019			2020		
	Q1	Q2	Q3	Q1	Q2	Q3	Q1	Q2	Q3
0.00	0.119	0.128	0.164	0.912	0.737	0.632	0.028	0.087	0.618
0.01 - 19.99	0.299	0.417	0.474	0.150	0.158	0.302	0.834	1.830	1.711
≥20.00	0.180	0.545	0.310	0.762	0.579	0.329	0.806	1.743	1.093

Table F.15 Bias Estimates of Prop. of German Citizens by Year and Quarter, BHP 2018-2020

Prop. of German Citizens	2018 Q1	2018 Q2	2018 Q3	2019 Q1	2019 Q2	2019 Q3	2020 Q1	2020 Q2	2020 Q3
100.00 (Ref.: 0.00-99.99)	0.551	0.966	0.626	0.497	0.459	0.672	0.096	1.229	1.036

Table F.16 Bias Estimates of Prop. of High-Educated Employees by Year and Quarter, BHP 2018-2020

Prop. of High-Educated Employees	2018 Q1	2018 Q2	2018 Q3	2019 Q1	2019 Q2	2019 Q3	2020 Q1	2020 Q2	2020 Q3
0.00	0.340	0.941	0.469	0.215	0.994	1.434	2.799	4.896	4.619
0.01 - 14.99	0.095	0.204	0.368	0.159	0.260	0.788	0.696	1.652	1.552
$\geq$15.00	0.244	0.738	0.837	0.055	0.734	0.646	2.103	3.243	3.067

Table F.17 Bias Estimates of Prop. of Low-Educated Employees by Year and Quarter, BHP 2018-2020

Prop. of Low-Educated Employees	2018 Q1	2018 Q2	2018 Q3	2019 Q1	2019 Q2	2019 Q3	2020 Q1	2020 Q2	2020 Q3
0.00	0.240	0.227	0.633	0.972	0.146	0.499	0.769	1.565	1.502
0.01 - 11.99	0.201	0.154	0.291	0.150	0.323	0.589	0.968	1.543	1.689
$\geq$12.00	0.039	0.074	0.925	1.122	0.470	0.090	0.199	0.022	0.187

Table F.18 Bias Estimates of Prop. of Mid-Educated Employees by Year and Quarter, BHP 2018-2020

Prop. of Mid-Educated Employees	2018 Q1	2018 Q2	2018 Q3	2019 Q1	2019 Q2	2019 Q3	2020 Q1	2020 Q2	2020 Q3
0.00 - 50.45	0.211	0.996	0.065	0.023	0.059	0.659	0.540	1.007	0.343
50.46 - 72.79	0.375	0.536	0.644	0.097	0.009	0.526	0.892	0.701	0.496
72.80 - 86.66	0.599	0.089	0.193	0.442	0.260	0.285	0.552	0.665	0.926
>86.67	0.435	0.370	0.386	0.516	0.210	0.153	1.984	2.372	1.765

Table F.19 Bias Estimates of Prop. of Unknown-Educated Employees by Year and Quarter, BHP 2018-2020

Prop. of Unknown-Educated Employees	2018 Q1	2018 Q2	2018 Q3	2019 Q1	2019 Q2	2019 Q3	2020 Q1	2020 Q2	2020 Q3
0.00 (Ref.: 0.01-100.00)	0.085	0.262	0.023	0.410	0.426	0.404	0.357	0.496	0.364

Table F.20 Bias Estimates of Prop. of Marginal Contracts by Year and Quarter, BHP 2018-2020

Prop. of Marginal Contracts	2018 Q1	2018 Q2	2018 Q3	2019 Q1	2019 Q2	2019 Q3	2020 Q1	2020 Q2	2020 Q3
0.00	0.679	0.355	0.818	0.156	0.344	0.890	0.204	0.147	0.244
0.00-14.99	0.034	0.371	0.128	0.165	0.657	0.539	0.832	1.605	1.659
$\geq$15.00	0.713	0.726	0.946	0.009	0.313	0.351	1.037	1.457	1.416

Table F.21 Bias Estimates of Prop. of Regular Contracts by Year and Quarter, BHP 2018-2020

Prop. of Regular Contracts	2018 Q1	2018 Q2	2018 Q3	2019 Q1	2019 Q2	2019 Q3	2020 Q1	2020 Q2	2020 Q3
0.00 - 70.73	1.148	0.025	1.192	0.009	0.198	0.053	1.312	1.726	2.427
70.74 - 87.57	0.757	0.699	0.167	0.295	0.031	0.427	0.598	0.873	1.755
87.58 - 96.93	0.080	0.213	0.265	0.083	0.454	0.412	0.775	1.050	1.135
>96.94	0.471	0.461	0.760	0.221	0.621	0.892	0.061	0.196	0.463

Table F.22 Bias Estimates of Quartile of Wage Distribution by Year and Quarter, BHP 2018-2020

Quartile of Wage Distribution	2018			2019			2020		
	Q1	Q2	Q3	Q1	Q2	Q3	Q1	Q2	Q3
First Quartile	0.419	0.330	0.122	0.731	0.163	0.701	0.352	0.501	0.925
Second Quartile	0.269	0.084	0.599	0.045	0.318	0.108	0.331	0.232	0.166
Third Quartile	0.160	0.009	0.127	0.077	0.478	0.521	0.564	1.256	0.275
Fourth Quartile	0.748	0.573	0.611	0.137	0.752	0.493	1.356	2.592	3.606
Missings	0.758	0.169	0.018	0.561	0.748	0.421	1.474	3.115	3.123

Table F.23 Bias Estimates of Short-Time Work Dummy by Year and Quarter, 2020

Short-Time Work Dummy	2018			2019			2020		
	Q1	Q2	Q3	Q1	Q2	Q3	Q1	Q2	Q3
No Short-Time Work (Ref.: Short-Time Work)	-	-	-	-	-	-	0.931	0.209	1.256

Appendix G: Survey Participation Models

G.1 Formulas

To address the third research question (RQ3), we run several logistic regression. The formulas behind these models are shown here.

Model 1: Baseline response model with core set of variables:

$$logit(R_{k,t}) = \alpha + \mathbf{x}_{1,k,t}^{\top}\boldsymbol{\beta}_1 + \mathbf{x}_{2,k,t}^{\top}\boldsymbol{\beta}_2 + \mathbf{z}_{1,k,t}^{\top}\boldsymbol{\gamma}_1 \tag{8}$$

where $logit(.)$ is the inverse of the standard logistic function, $R_{k,t}$ is the response indicator for the kth establishment ($R_k = 1 =$ response, $R_k = 0 =$ nonresponse) in survey quarter t, $\mathbf{x}_1$ includes administrative variables of interest (industry, establishment size), $\mathbf{x}_2$ includes paradata (early vs. late respondents, contact person provided, telephone number provided), and $\mathbf{z}_1$ is a set of administrative control variables (foundation year, establishment founded in sampling year, federal state, inhabitants in a municipality). α represents the intercept, $\boldsymbol{\beta}_1$ defines the corresponding vector of coefficients for $\mathbf{x}_1$, $\boldsymbol{\beta}_2$ defines the corresponding vector of coefficients for $\mathbf{x}_2$, and $\boldsymbol{\gamma}_1$ defines the corresponding vector of coefficients for $\mathbf{z}_1$.

Model 2: Response model with COVID-19 variables:

$$logit(R_{k,t}) = \alpha + \mathbf{w}_{1,k,t}^{\top}\boldsymbol{\eta}_1 \tag{9}$$

where $\mathbf{w}_1$ denotes COVID-19 specific variables.

Model 3: Response model with COVID-19 variables and core set of variables:

$$logit(R_{k,t}) = \alpha + \mathbf{w}_{1,k,t}^{\top}\boldsymbol{\eta}_1 + \mathbf{x}_{1,k,t}^{\top}\boldsymbol{\beta}_1 + \mathbf{x}_{2,k,t}^{\top}\boldsymbol{\beta}_2 + \mathbf{z}_{1,k,t}^{\top}\boldsymbol{\gamma}_1 \tag{10}$$

where $\mathbf{w}_1$ denotes COVID-19 specific variables, $\mathbf{x}_1$ denotes administrative variables of interest, $\mathbf{x}_2$ denotes paradata, and $\mathbf{z}_1$ is a set of administrative control variables.

Model 4: Response model with COVID-19 variables and core set of variables with interactions of short-time work with establishment size and industry:

$$logit(R_{k,t}) = \alpha + \mathbf{w}_{1,k,t}^{\top}\boldsymbol{\eta}_1 + \mathbf{x}_{1,k,t}^{\top}\boldsymbol{\beta}_1 + \mathbf{x}_{2,k,t}^{\top}\boldsymbol{\beta}_2 + \mathbf{x}_{3,k,t}^{\top}\boldsymbol{\beta}_3 + \mathbf{z}_{1,k,t}^{\top}\boldsymbol{\gamma}_1 \tag{11}$$

where $\mathbf{w}_1$ denotes COVID-19 specific variables, $\mathbf{x}_1$ denotes administrative variables of interest, $\mathbf{x}_2$ denotes paradata, $\mathbf{x}_3$ denotes interaction terms of short-time work with establishment size and industry, and $\mathbf{z}_1$ is a set of administrative control variables.

Table G.1 Baseline Survey Participation Model by Quarter and Year, 2018-2020

	Quarter 1						Quarter 2						Quarter 3					
	Odds Ratio (Std. Err.)			Sig. Diff.			Odds Ratio (Std. Err.)			Sig. Diff.			Odds Ratio (Std. Err.)			Sig. Diff.		
	2018	2019	2020	18 vs. 19	18 vs. 20	19 vs. 20	2018	2019	2020	18 vs. 19	18 vs. 20	19 vs. 20	2018	2019	2020	18 vs. 19	18 vs. 20	19 vs. 20
DV: Response																		
Industry (Ref.: Agriculture/Production)																		
Energy/Construction/Logistic	0.78	0.70	0.98	-	-	-	0.82	0.63*	0.74**	-	-	-	0.78	0.96	0.70**	-	-	-
	(0.19)	(0.16)	(0.13)				(0.19)	(0.12)	(0.08)				(0.27)	(0.21)	(0.07)			
Retail/Hospitality/Entertainment	0.68	0.81	0.89	-	-	-	0.65	0.73	0.83	-	-	-	0.80	0.70	0.77*	-	-	-
	(0.15)	(0.22)	(0.14)				(0.15)	(0.16)	(0.10)				(0.26)	(0.15)	(0.10)			
Finance/Information/Real Estate	0.67	0.91	0.89	-	-	-	1.55	0.79	0.89	*	*	-	1.17	0.72	1.09	-	-	-
	(0.15)	(0.25)	(0.13)				(0.37)	(0.17)	(0.10)				(0.40)	(0.16)	(0.13)			
Public/Educ./Health	1.63	0.62	0.85	**	*	-	1.04	1.35	0.75*	-	-	*	0.67	1.56	0.74*	*	-	*
	(0.43)	(0.15)	(0.14)				(0.27)	(0.34)	(0.09)				(0.22)	(0.42)	(0.09)			
Other Services	0.71	0.86	0.91	-	-	-	0.89	0.63*	0.98	-	-	-	1.24	0.70	1.01	-	-	-
	(0.18)	(0.24)	(0.15)				(0.22)	(0.14)	(0.13)				(0.47)	(0.17)	(0.13)			
Number of Employees (Ref.: 1-9)																		
10-19	1.09	1.24	1.28	-	-	-	1.12	1.05	1.39**	-	-	-	1.00	1.21	1.16	-	-	-
	(0.20)	(0.23)	(0.17)				(0.23)	(0.21)	(0.15)				(0.27)	(0.23)	(0.13)			
20-49	1.06	1.08	1.17	-	-	-	1.35	1.25	1.43***	-	-	-	1.47	1.55*	1.47***	-	-	-
	(0.18)	(0.19)	(0.13)				(0.25)	(0.21)	(0.12)				(0.34)	(0.30)	(0.13)			
50-249	0.96	1.88**	1.50**	*	*	-	1.37	1.50*	1.52***	-	-	-	1.37	1.49*	1.77***	-	-	-
	(0.18)	(0.42)	(0.20)				(0.26)	(0.26)	(0.16)				(0.35)	(0.27)	(0.18)			
≥250	1.22	2.41**	1.39*	-	-	-	1.67*	1.43	1.29*	-	-	-	1.73	1.70*	1.23	-	-	-
	(0.28)	(0.70)	(0.21)				(0.40)	(0.32)	(0.15)				(0.49)	(0.40)	(0.14)			
Foundation Year (Ref.: 70s/80s)																		
90s	0.87	0.88	0.93	-	-	-	1.03	1.35	0.97	-	-	-	0.56	1.43	0.80	*	-	*
	(0.22)	(0.21)	(0.15)				(0.27)	(0.30)	(0.12)				(0.17)	(0.33)	(0.10)			
00s	1.26	1.37	1.09	-	-	-	0.96	1.10	1.07	-	-	-	1.96*	1.12	0.91	-	*	-
	(0.29)	(0.33)	(0.16)				(0.23)	(0.23)	(0.13)				(0.63)	(0.25)	(0.11)			
10s	1.44	1.05	0.97	-	-	-	1.46	1.10	0.91	-	-	-	0.79	1.10	0.68**	-	-	-
	(0.38)	(0.26)	(0.15)				(0.43)	(0.26)	(0.11)				(0.27)	(0.27)	(0.09)			
Federal State aggregated																		
(Ref.: Schleswig-Holstein + Hamburg)																		
Lower Saxony + Bremen	0.52	0.51	1.19	-	-	-	0.78	1.36	0.98	-	-	-	0.41	1.21	1.57*	-	*	-
	(0.23)	(0.23)	(0.30)				(0.36)	(0.52)	(0.21)				(0.22)	(0.44)	(0.35)			
North Rhine-Westphalia	0.48	0.46*	1.10	-	-	*	0.88	0.92	0.75	-	-	-	0.37*	1.15	1.20	-	*	-
	(0.20)	(0.17)	(0.27)				(0.37)	(0.32)	(0.15)				(0.18)	(0.39)	(0.24)			
Hesse	0.86	0.99	0.69	-	-	-	0.53	1.17	0.78	-	-	-	1.39	2.16	1.23	-	-	-
	(0.44)	(0.44)	(0.19)				(0.25)	(0.50)	(0.19)				(0.92)	(0.92)	(0.29)			
Rhineland-Palatinate + Saarland	0.43	0.36*	0.71	-	-	-	0.97	1.44	0.84	-	-	-	0.38	2.01	1.77*	*	*	-
	(0.23)	(0.17)	(0.20)				(0.56)	(0.62)	(0.21)				(0.24)	(0.89)	(0.44)			
Baden-Wuerttemberg	0.80	0.85	1.30	-	-	-	1.66	2.56**	0.78	-	-	**	1.12	1.68	1.15	-	-	-
	(0.36)	(0.36)	(0.32)				(0.74)	(0.92)	(0.16)				(0.64)	(0.65)	(0.24)			
Bavaria	0.75	1.05	1.91**	-	-	-	1.12	1.47	1.21	-	-	-	0.78	2.64**	1.65*	-	-	-
	(0.32)	(0.43)	(0.45)				(0.49)	(0.53)	(0.25)				(0.41)	(0.97)	(0.33)			
Brandenburg + Berlin	0.75	1.00	1.33	-	-	-	1.00	1.26	0.79	-	-	-	1.04	1.91	1.07	-	-	-
	(0.33)	(0.41)	(0.37)				(0.44)	(0.47)	(0.18)				(0.56)	(0.80)	(0.24)			
Mecklenburg-Vorpommern	0.30*	0.60	1.31	-	*	-	0.61	0.93	0.53*	-	-	-	0.49	1.40	0.65	-	-	-
	(0.17)	(0.35)	(0.52)				(0.31)	(0.48)	(0.15)				(0.34)	(0.72)	(0.19)			
Saxony	0.53	1.13	1.19	-	-	-	1.40	2.36*	1.05	-	-	-	2.32	1.38	1.46	-	-	-
	(0.24)	(0.52)	(0.35)				(0.64)	(0.96)	(0.25)				(1.26)	(0.58)	(0.36)			
Saxony-Anhalt	1.42	0.90	0.86	-	-	-	1.19	0.98	0.76	-	-	-	0.59	1.25	1.41	-	-	-
	(0.81)	(0.50)	(0.32)				(0.65)	(0.49)	(0.22)				(0.37)	(0.60)	(0.41)			
Thuringia	0.70	0.90	1.46	-	-	-	1.01	1.11	0.97	-	-	-	1.50	2.30	1.38	-	-	-
	(0.39)	(0.52)	(0.55)				(0.57)	(0.57)	(0.27)				(0.91)	(1.25)	(0.40)			
Inhabitants in Municipality (Ref.: <15,000)																		
15,000-99,999	0.82	1.29	1.10	-	-	-	0.90	0.82	0.90	-	-	-	0.57*	1.22	1.06	*	*	-
	(0.16)	(0.26)	(0.14)				(0.18)	(0.15)	(0.09)				(0.16)	(0.24)	(0.11)			
≥100,000	1.13	1.09	1.01	-	-	-	1.08	0.84	0.91	-	-	-	0.79	1.72*	1.07	*	-	-
	(0.24)	(0.26)	(0.14)				(0.26)	(0.19)	(0.10)				(0.22)	(0.40)	(0.12)			
Establishment Founded in Sampling Year	1.45	5.83**	1.06	-	-	*	0.56	0.70	1.20	-	-	-	0.52	1.07	0.89	-	-	-
	(0.84)	(3.86)	(0.32)				(0.30)	(0.30)	(0.31)				(0.32)	(0.52)	(0.23)			
50 Earliest Percent of Respondents in Q4	1.05	1.01	1.29*	-	-	-	1.28	1.15	1.32**	-	-	-	1.52	1.37*	1.14	-	-	-
	(0.17)	(0.18)	(0.14)				(0.21)	(0.18)	(0.11)				(0.35)	(0.21)	(0.10)			
Contact Person Provided in Q4	1.21	1.10	1.24	-	-	-	1.07	1.96	1.55	-	-	-	1.05	0.65	1.07	-	-	-
	(0.32)	(0.53)	(0.41)				(0.29)	(0.95)	(0.38)				(0.33)	(0.28)	(0.28)			
Telephone Number Provided in Q4	2.95***	4.52**	1.28	-	*	*	2.42**	0.69	1.16	*	*	-	1.92*	1.97	1.10	-	-	-
	(0.72)	(2.08)	(0.37)				(0.62)	(0.31)	(0.24)				(0.58)	(0.73)	(0.25)			
Constant	5.52***	3.24**	1.50				3.49**	4.16***	0.88				15.53***	2.25	1.13			
	(2.51)	(1.45)	(0.42)				(1.58)	(1.62)	(0.21)				(9.89)	(0.98)	(0.28)			
N (unweighted)	9,845	9,740	10,664				9,769	9,916	13,122				9,422	9,817	12,794			

NOTES: Odds Ratios; Standard errors in parentheses

+ $p < 0.10$, * $p < 0.05$, ** $p < 0.01$, *** $p < 0.001$

Weighted for Inclusion Probability, Response Propensity in the First Wave (Q4) and Fielding Probability.

Appendix H: Nonresponse Bias Adjustments

H.1 COVID-19 Weighting Schemes

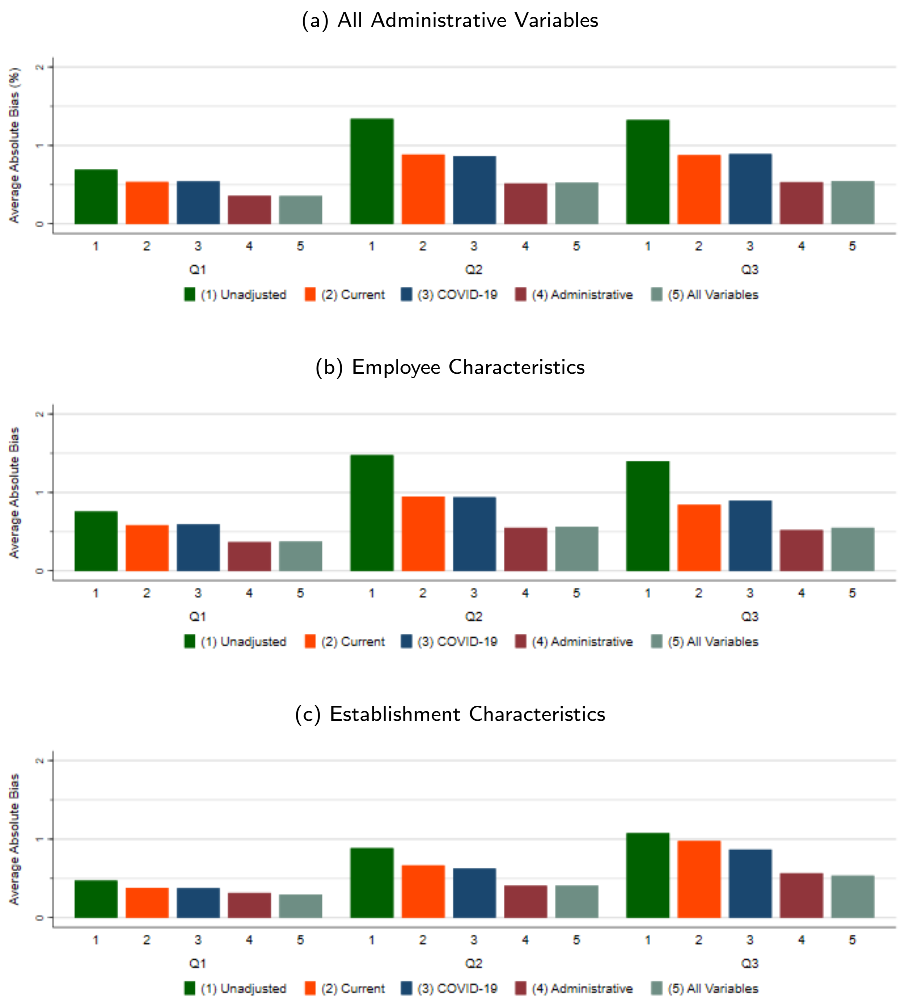

Figure H.1 Average Absolute Nonresponse Bias by Weighting Scheme, BHP 2020

H.2 Average Absolute Bias

Table H.1 Average Absolute Bias by Set of Explanatory Variables, All Administrative Variables BHP 2020

	Q1	Q2	Q3
Unadjusted	0.690	1.338	1.325
Current	0.535	0.883	0.875
COVID-19	0.541	0.866	0.890
Administrative	0.356	0.517	0.532
All Variables	0.355	0.527	0.546

Table H.2 Average Absolute Bias by Set of Explanatory Variables, Establishment Characteristics BHP 2020

	Q1	Q2	Q3
Unadjusted	0.472	0.885	1.076
Current	0.378	0.666	0.975
COVID-19	0.373	0.625	0.867
Administrative	0.315	0.407	0.567
All Variables	0.294	0.410	0.534

Table H.3 Average Absolute Bias by Set of Explanatory Variables, Employee Characteristics BHP 2020

	Q1	Q2	Q3
Unadjusted	0.757	1.475	1.400
Current	0.582	0.949	0.844
COVID-19	0.592	0.938	0.897
Administrative	0.369	0.550	0.521
All Variables	0.374	0.562	0.549

Table H.4 Average Absolute Bias by Set of Explanatory Variables, All Administrative Variables BHP 2018 - 2020

	Q1			Q2			Q3		
	2018	2019	2020	2018	2019	2020	2018	2019	2020
Unadjusted	0.352	0.291	0.690	0.384	0.403	1.338	0.362	0.480	1.325
Current	0.345	0.304	0.535	0.348	0.344	0.883	0.358	0.337	0.875
Administrative	0.305	0.270	0.356	0.301	0.239	0.517	0.245	0.259	0.532

Table H.5 Average Absolute Bias by Set of Explanatory Variables, Establishment Characteristics BHP 2018 - 2020

	Q1			Q2			Q3		
	2018	2019	2020	2018	2019	2020	2018	2019	2020
Unadjusted	0.303	0.248	0.472	0.239	0.354	0.885	0.262	0.432	1.076
Current	0.316	0.260	0.378	0.232	0.340	0.666	0.255	0.395	0.975
Administrative	0.338	0.254	0.315	0.263	0.252	0.407	0.307	0.254	0.567

Table H.6 Average Absolute Bias by Set of Explanatory Variables, Employee Characteristics BHP 2018 - 2020

	Q1			Q2			Q3		
	2018	2019	2020	2018	2019	2020	2018	2019	2020
Unadjusted	0.367	0.305	0.757	0.428	0.418	1.475	0.392	0.494	1.400
Current	0.354	0.318	0.582	0.383	0.345	0.949	0.389	0.319	0.844
Administrative	0.294	0.274	0.369	0.313	0.235	0.550	0.226	0.260	0.521

H.3 Single Absolute Bias Estimates

Table H.7 Bias Estimates of Foundation Year by Quarter and Weighting Strategy, BHP 2018-2020

Variable	Unadjusted	Current	COVID-19	Admin	All
Quarter 1 - 2018					
70s/80s	0.058	0.173	-	0.245	-
90s	0.634	0.655	-	0.640	-
00s	0.186	0.237	-	0.451	-
10s	0.506	0.590	-	0.434	-
Quarter 1 - 2019					
70s/80s	0.102	0.198	-	0.030	-
90s	0.297	0.302	-	0.261	-
00s	0.554	0.610	-	0.513	-
10s	0.155	0.110	-	0.221	-
Quarter 1 - 2020					
70s/80s	0.338	0.013	0.113	0.014	0.023
90s	0.258	0.307	0.259	0.551	0.555
00s	0.506	0.575	0.572	0.580	0.563
10s	0.586	0.255	0.199	0.042	0.030
Quarter 2 - 2018					
70s/80s	0.093	0.089	-	0.081	-
90s	0.034	0.010	-	0.039	-
00s	0.573	0.535	-	0.587	-
10s	0.700	0.614	-	0.545	-
Quarter 2 - 2019					
70s/80s	0.039	0.318	-	0.316	-
90s	0.671	0.681	-	0.546	-
00s	0.205	0.080	-	0.146	-
10s	0.427	0.283	-	0.084	-
Quarter 2 - 2020					
70s/80s	0.990	0.310	0.184	0.150	0.102
90s	0.013	0.089	0.060	0.281	0.250
00s	0.523	0.670	0.780	0.627	0.718
10s	1.526	0.891	0.904	0.496	0.571
Quarter 3 - 2018					
70s/80s	0.007	0.051	-	0.011	-
90s	0.739	0.747	-	0.898	-
00s	1.152	1.168	-	1.309	-
10s	0.420	0.370	-	0.400	-
Quarter 3 - 2019					
70s/80s	0.077	0.391	-	0.382	-
90s	0.544	0.601	-	0.579	-
00s	0.205	0.129	-	0.111	-
10s	0.261	0.081	-	0.086	-
Quarter 3 - 2020					
70s/80s	2.153	1.522	1.474	1.019	1.095
90s	0.342	0.319	0.135	0.469	0.455
00s	0.839	1.064	0.994	1.089	1.064
10s	2.651	2.267	2.333	1.638	1.704

Table H.8 Bias Estimates of Number of Employees by Quarter and Weighting Strategy, BHP 2018-2020

Variable	Unadjusted	Current	COVID-19	Admin	All
Quarter 1 - 2018					
1-9	0.164	0.023	-	0.056	-
10-19	0.136	0.110	-	0.194	-
20-49	0.002	0.050	-	0.069	-
≥50	0.026	0.037	-	0.069	-
Quarter 1 - 2019					
1-9	0.289	0.285	-	0.261	-
10-19	0.184	0.199	-	0.157	-
20-49	0.114	0.107	-	0.086	-
≥50	0.220	0.193	-	0.189	-
Quarter 1 - 2020					
1-9	1.306	0.970	0.961	0.042	0.053
10-19	0.589	0.489	0.463	0.104	0.079
20-49	0.235	0.134	0.137	0.153	0.143
≥50	0.482	0.348	0.361	0.092	0.116
Quarter 2 - 2018					
1-9	0.507	0.528	-	0.219	-
10-19	0.087	0.159	-	0.299	-
20-49	0.228	0.234	-	0.014	-
≥50	0.192	0.134	-	0.094	-
Quarter 2 - 2019					
1-9	0.504	0.489	-	0.132	-
10-19	0.006	0.027	-	0.214	-
20-49	0.207	0.222	-	0.011	-
≥50	0.291	0.240	-	0.071	-
Quarter 2 - 2020					
1-9	3.160	2.473	2.446	0.489	0.560
10-19	1.358	1.215	1.272	0.570	0.645
20-49	1.000	0.803	0.762	0.093	0.094
≥50	0.802	0.454	0.412	0.174	0.179
Quarter 3 - 2018					
1-9	0.098	0.007	-	0.408	-
10-19	0.122	0.125	-	0.038	-
20-49	0.138	0.092	-	0.308	-
≥50	0.082	0.040	-	0.062	-
Quarter 3 - 2019					
1-9	0.937	0.790	-	0.080	-
10-19	0.240	0.217	-	0.058	-
20-49	0.409	0.370	-	0.052	-
≥50	0.288	0.203	-	0.075	-
Quarter 3 - 2020					
1-9	2.828	2.396	2.183	0.040	0.008
10-19	0.425	0.446	0.327	0.282	0.320
20-49	1.189	1.051	1.030	0.207	0.241
≥50	1.214	0.898	0.826	0.114	0.071

Table H.9 Bias Estimates of Industry by Quarter and Weighting Strategy, BHP 2018-2020

Variable	Unadjusted	Current	COVID-19	Admin	All
Quarter 1 - 2018					
Agric./Production	0.246	0.238	-	0.119	-
Service	1.037	1.034	-	1.065	-
Public/Educ./Health/Arts	0.791	0.795	-	0.946	-
Quarter 1 - 2019					
Agric./Production	0.040	0.024	-	0.213	-
Service	0.265	0.296	-	0.172	-
Public/Educ./Health/Arts	0.305	0.320	-	0.385	-
Quarter 1 - 2020					
Agric./Production	0.892	0.838	0.642	0.865	0.752
Service	0.799	0.670	0.516	1.148	1.028
Public/Educ./Health/Arts	0.093	0.167	0.126	0.283	0.277
Quarter 2 - 2018					
Agric./Production	0.001	0.010	-	0.247	-
Service	0.226	0.207	-	0.479	-
Public/Educ./Health/Arts	0.227	0.217	-	0.232	-
Quarter 2 - 2019					
Agric./Production	0.358	0.330	-	0.502	-
Service	0.935	0.842	-	0.772	-
Public/Educ./Health/Arts	0.577	0.513	-	0.270	-
Quarter 2 - 2020					
Agric./Production	0.614	0.450	0.119	0.027	0.091
Service	0.393	0.097	0.145	0.668	0.566
Public/Educ./Health/Arts	0.221	0.353	0.264	0.642	0.656
Quarter 3 - 2018					
Agric./Production	0.063	0.067	-	0.026	-
Service	0.004	0.011	-	0.014	-
Public/Educ./Health/Arts	0.060	0.055	-	0.011	-
Quarter 3 - 2019					
Agric./Production	0.202	0.170	-	0.265	-
Service	1.176	1.074	-	0.884	-
Public/Educ./Health/Arts	0.975	0.903	-	0.619	-
Quarter 3 - 2020					
Agric./Production	0.090	0.157	0.152	0.567	0.539
Service	0.165	0.557	0.470	0.065	0.024
Public/Educ./Health/Arts	0.255	0.400	0.317	0.501	0.563

Table H.10 Bias Estimates of Establishment Founded in Sampling Year by Quarter and Weighting Strategy, BHP 2018-2020

Variable	Unadjusted	Current	COVID-19	Admin	All
Quarter 1 - 2018					
Founded Before Sampling Year (Ref.: Founded in Sampling Year)	0.110	0.114	-	0.071	-
Quarter 1 - 2019					
Founded Before Sampling Year (Ref.: Founded in Sampling Year)	0.223	0.234	-	0.231	-
Quarter 1 - 2020					
Founded Before Sampling Year (Ref.: Founded in Sampling Year)	0.039	0.018	0.037	0.024	0.020
Quarter 2 - 2018					
Founded Before Sampling Year (Ref.: Founded in Sampling Year)	0.163	0.151	-	0.249	-
Quarter 2 - 2019					
Founded Before Sampling Year (Ref.: Founded in Sampling Year)	0.234	0.209	-	0.196	-
Quarter 2 - 2020					
Founded Before Sampling Year (Ref.: Founded in Sampling Year)	0.019	0.130	0.176	0.120	0.151
Quarter 3 - 2018					
Founded Before Sampling Year (Ref.: Founded in Sampling Year)	0.145	0.150	-	0.149	-
Quarter 3 - 2019					
Founded Before Sampling Year (Ref.: Founded in Sampling Year)	0.065	0.040	-	0.017	-
Quarter 3 - 2020					
Founded Before Sampling Year (Ref.: Founded in Sampling Year)	0.382	0.274	0.312	0.127	0.170

Table H.11 Bias Estimates of East/West Germany by Quarter and Weighting Strategy, BHP 2018-2020

Variable	Unadjusted	Current	COVID-19	Admin	All
Quarter 1 - 2018					
East Germany (Ref.: West Germany)	0.047	0.053	-	0.041	-
Quarter 1 - 2019					
East Germany (Ref.: West Germany)	0.470	0.501	-	0.585	-
Quarter 1 - 2020					
East Germany (Ref.: West Germany)	0.010	0.135	0.459	0.197	0.179
Quarter 2 - 2018					
East Germany (Ref.: West Germany)	0.070	0.130	-	0.332	-
Quarter 2 - 2019					
East Germany (Ref.: West Germany)	0.146	0.186	-	0.022	-
Quarter 2 - 2020					
East Germany (Ref.: West Germany)	0.890	0.725	0.607	0.952	0.747
Quarter 3 - 2018					
East Germany (Ref.: West Germany)	0.379	0.433	-	0.357	-
Quarter 3 - 2019					
East Germany (Ref.: West Germany)	0.238	0.172	-	0.097	-
Quarter 3 - 2020					
East Germany (Ref.: West Germany)	1.460	1.326	0.716	1.250	0.692

Table H.12 Bias Estimates of Avg. Age of Employees by Quarter and Weighting Strategy, BHP 2018-2020

Variable	Unadjusted	Current	COVID-19	Admin	All
Quarter 1 - 2018					
0.00-38.99	0.349	0.344	-	0.298	-
39.00-43.49	0.125	0.108	-	0.239	-
43.50-47.99	0.163	0.121	-	0.101	-
$\geq$48.00	0.387	0.357	-	0.161	-
Quarter 1 - 2019					
0.00-38.99	0.254	0.289	-	0.548	-
39.00-43.49	0.220	0.258	-	0.136	-
43.50-47.99	0.466	0.506	-	0.532	-
$\geq$48.00	0.432	0.475	-	0.120	-
Quarter 1 - 2020					
0.00-38.99	0.346	0.398	0.297	0.371	0.317
39.00-43.49	0.162	0.287	0.273	0.400	0.402
43.50-47.99	0.778	0.592	0.563	0.505	0.501
$\geq$48.00	0.270	0.093	0.007	0.266	0.218
Quarter 2 - 2018					
0.00-38.99	0.280	0.332	-	0.264	-
39.00-43.49	0.404	0.377	-	0.188	-
43.50-47.99	0.300	0.283	-	0.307	-
$\geq$48.00	0.984	0.991	-	0.759	-
Quarter 2 - 2019					
0.00-38.99	0.056	0.081	-	0.085	-
39.00-43.49	0.535	0.435	-	0.376	-
43.50-47.99	0.303	0.212	-	0.157	-
$\geq$48.00	0.782	0.566	-	0.618	-
Quarter 2 - 2020					
0.00-38.99	0.661	0.418	0.392	0.637	0.683
39.00-43.49	0.118	0.407	0.380	0.549	0.484
43.50-47.99	0.269	0.125	0.148	0.373	0.383
$\geq$48.00	0.511	0.951	0.920	1.559	1.550
Quarter 3 - 2018					
0.00-38.99	0.250	0.198	-	0.227	-
39.00-43.49	0.143	0.125	-	0.095	-
43.50-47.99	0.641	0.642	-	0.678	-
$\geq$48.00	0.749	0.715	-	0.356	-
Quarter 3 - 2019					
0.00-38.99	0.548	0.504	-	0.283	-
39.00-43.49	0.589	0.445	-	0.262	-
43.50-47.99	0.784	0.654	-	0.548	-
$\geq$48.00	0.824	0.594	-	0.526	-
Quarter 3 - 2020					
0.00-38.99	0.619	0.326	0.511	0.006	0.179
39.00-43.49	0.071	0.278	0.264	0.592	0.581
43.50-47.99	0.399	0.069	0.033	0.457	0.417
$\geq$48.00	0.149	0.673	0.808	1.056	1.177

Table H.13 Bias Estimates of Prop. of Apprentices by Quarter and Weighting Strategy, BHP 2018-2020

Variable	Unadjusted	Current	COVID-19	Admin	All
Quarter 1 - 2018					
0.00 (Ref.: 0.01-100)	0.056	0.128	-	0.180	-
Quarter 1 - 2019					
0.00 (Ref.: 0.01-100)	0.006	0.142	-	0.283	-
Quarter 1 - 2020					
0.00 (Ref.: 0.01-100)	0.776	0.126	0.078	0.256	0.209
Quarter 2 - 2018					
0.00 (Ref.: 0.01-100)	0.521	0.357	-	0.275	-
Quarter 2 - 2019					
0.00 (Ref.: 0.01-100)	0.765	0.426	-	0.265	-
Quarter 2 - 2020					
0.00 (Ref.: 0.01-100)	1.740	0.605	0.630	0.567	0.583
Quarter 3 - 2018					
0.00 (Ref.: 0.01-100)	0.105	0.180	-	0.146	-
Quarter 3 - 2019					
0.00 (Ref.: 0.01-100)	0.550	0.144	-	0.147	-
Quarter 3 - 2020					
0.00 (Ref.: 0.01-100)	2.033	1.028	1.079	0.975	1.062

Table H.14 Bias Estimates of Prop. of Female Employees by Quarter and Weighting Strategy, BHP 2018-2020

Variable	Unadjusted	Current	COVID-19	Admin	All
Quarter 1 - 2018					
0.00 - 18.18	0.205	0.151	-	0.014	-
18.19 - 41.93	0.405	0.390	-	0.363	-
41.94 - 68.38	0.717	0.723	-	0.927	-
>68.39	1.327	1.264	-	1.276	-
Quarter 1 - 2019					
0.00 - 18.18	0.393	0.413	-	0.288	-
18.19 - 41.93	0.216	0.155	-	0.191	-
41.94 - 68.38	0.300	0.340	-	0.394	-
>68.39	0.477	0.598	-	0.491	-
Quarter 1 - 2020					
0.00 - 18.18	0.150	0.308	0.327	0.349	0.385
18.19 - 41.93	0.918	0.662	0.730	0.606	0.678
41.94 - 68.38	0.435	0.402	0.495	0.389	0.503
>68.39	1.203	0.756	0.898	0.646	0.797
Quarter 2 - 2018					
0.00 - 18.18	0.454	0.437	-	0.495	-
18.19 - 41.93	0.163	0.135	-	0.026	-
41.94 - 68.38	0.945	0.824	-	0.797	-
>68.39	0.328	0.252	-	0.276	-
Quarter 2 - 2019					
0.00 - 18.18	0.396	0.243	-	0.112	-
18.19 - 41.93	0.126	0.050	-	0.191	-
41.94 - 68.38	0.393	0.354	-	0.172	-
>68.39	0.129	0.062	-	0.093	-
Quarter 2 - 2020					
0.00 - 18.18	0.766	0.869	0.758	0.958	0.844
18.19 - 41.93	2.134	1.635	1.678	1.163	1.223
41.94 - 68.38	1.020	0.854	0.999	0.702	0.826
>68.39	3.919	3.358	3.435	2.823	2.894
Quarter 3 - 2018					
0.00 - 18.18	0.296	0.173	-	0.168	-
18.19 - 41.93	0.096	0.022	-	0.109	-
41.94 - 68.38	0.207	0.254	-	0.070	-
>68.39	0.185	0.103	-	0.206	-
Quarter 3 - 2019					
0.00 - 18.18	0.172	0.113	-	0.231	-
18.19 - 41.93	0.092	0.100	-	0.328	-
41.94 - 68.38	0.093	0.119	-	0.046	-
>68.39	0.171	0.095	-	0.051	-
Quarter 3 - 2020					
0.00 - 18.18	0.204	0.355	0.443	0.535	0.627
18.19 - 41.93	1.871	1.528	1.455	0.944	0.863
41.94 - 68.38	1.106	0.658	0.596	0.660	0.616
>68.39	3.181	2.541	2.494	2.139	2.106

Table H.15 Bias Estimates of Prop. of Fixed-Term Contracts by Quarter and Weighting Strategy, BHP 2018-2020

Variable	Unadjusted	Current	COVID-19	Admin	All
Quarter 1 - 2018					
0.00	0.697	0.565	-	0.376	-
0.01 - 15.99	0.193	0.168	-	0.166	-
≥16.00	0.504	0.397	-	0.210	-
Quarter 1 - 2019					
0.00	0.120	0.235	-	0.247	-
0.01 - 15.99	0.046	0.058	-	0.110	-
≥16.00	0.166	0.177	-	0.137	-
Quarter 1 - 2020					
0.00	0.369	0.428	0.420	0.567	0.530
0.01 - 15.99	0.792	0.245	0.271	0.226	0.275
≥16.00	0.423	0.673	0.691	0.792	0.805
Quarter 2 - 2018					
0.00	1.037	0.802	-	0.568	-
0.01 - 15.99	0.539	0.391	-	0.360	-
≥16.00	0.499	0.412	-	0.208	-
Quarter 2 - 2019					
0.00	0.997	0.475	-	0.277	-
0.01 - 15.99	0.443	0.230	-	0.186	-
≥16.00	0.554	0.244	-	0.090	-
Quarter 2 - 2020					
0.00	2.836	1.189	1.184	0.931	0.945
0.01 - 15.99	1.525	0.377	0.362	0.261	0.266
≥16.00	1.311	0.813	0.822	0.669	0.679
Quarter 3 - 2018					
0.00	0.216	0.272	-	0.058	-
0.01 - 15.99	0.051	0.138	-	0.059	-
≥16.00	0.165	0.133	-	0.118	-
Quarter 3 - 2019					
0.00	1.036	0.379	-	0.107	-
0.01 - 15.99	0.127	0.219	-	0.326	-
≥16.00	0.908	0.597	-	0.433	-
Quarter 3 - 2020					
0.00	2.369	0.702	0.817	0.275	0.365
0.01 - 15.99	1.793	0.658	0.654	0.506	0.499
≥16.00	0.576	0.044	0.163	0.231	0.134

Table H.16 Bias Estimates of Prop. of Full-Time Contracts by Quarter and Weighting Strategy, BHP 2018-2020

Variable	Unadjusted	Current	COVID-19	Admin	All
Quarter 1 - 2018					
0.00 - 34.40	0.039	0.007	-	0.782	-
34.41 - 65.35	0.172	0.228	-	0.390	-
65.36 - 85.15	0.017	0.004	-	0.328	-
>85.16	0.194	0.216	-	0.064	-
Quarter 1 - 2019					
0.00 - 34.40	0.248	0.423	-	0.241	-
34.41 - 65.35	0.131	0.054	-	0.161	-
65.36 - 85.15	0.134	0.088	-	0.242	-
>85.16	0.513	0.566	-	0.162	-
Quarter 1 - 2020					
0.00 - 34.40	1.694	1.132	1.190	0.087	0.093
34.41 - 65.35	0.712	0.579	0.558	0.417	0.400
65.36 - 85.15	0.395	0.005	0.011	0.417	0.412
>85.16	0.588	0.558	0.621	0.086	0.105
Quarter 2 - 2018					
0.00 - 34.40	0.404	0.617	-	0.665	-
34.41 - 65.35	0.391	0.574	-	0.530	-
65.36 - 85.15	0.250	0.258	-	0.081	-
>85.16	0.238	0.215	-	0.054	-
Quarter 2 - 2019					
0.00 - 34.40	0.272	0.278	-	0.026	-
34.41 - 65.35	0.806	0.746	-	0.201	-
65.36 - 85.15	0.045	0.019	-	0.003	-
>85.16	0.489	0.449	-	0.230	-
Quarter 2 - 2020					
0.00 - 34.40	4.410	3.335	3.319	1.264	1.307
34.41 - 65.35	2.092	1.628	1.622	1.137	1.133
65.36 - 85.15	2.035	1.361	1.354	0.630	0.655
>85.16	0.283	0.346	0.342	0.503	0.481
Quarter 3 - 2018					
0.00 - 34.40	0.147	0.375	-	0.564	-
34.41 - 65.35	0.790	0.840	-	0.631	-
65.36 - 85.15	0.072	0.011	-	0.028	-
>85.16	0.571	0.476	-	0.096	-
Quarter 3 - 2019					
0.00 - 34.40	0.363	0.250	-	0.345	-
34.41 - 65.35	0.520	0.460	-	0.356	-
65.36 - 85.15	0.256	0.161	-	0.078	-
>85.16	0.412	0.371	-	0.088	-
Quarter 3 - 2020					
0.00 - 34.40	3.835	2.833	2.955	0.820	0.940
34.41 - 65.35	1.430	1.086	1.092	0.322	0.354
65.36 - 85.15	1.990	1.408	1.406	0.604	0.659
>85.16	0.415	0.339	0.456	0.106	0.073

Table H.17 Bias Estimates of Prop. of German Citizens by Quarter and Weighting Strategy, BHP 2018-2020

Variable	Unadjusted	Current	COVID-19	Admin	All
Quarter 1 - 2018					
100.00 (Ref.: 0.00-99.99)	0.551	0.492	-	0.433	-
Quarter 1 - 2019					
100.00 (Ref.: 0.00-99.99)	0.497	0.577	-	0.302	-
Quarter 1 - 2020					
100.00 (Ref.: 0.00-99.99)	0.096	0.751	0.695	0.701	0.684
Quarter 2 - 2018					
100.00 (Ref.: 0.00-99.99)	0.966	0.841	-	0.790	-
Quarter 2 - 2019					
100.00 (Ref.: 0.00-99.99)	0.459	0.675	-	0.426	-
Quarter 2 - 2020					
100.00 (Ref.: 0.00-99.99)	1.229	0.061	0.050	0.055	0.071
Quarter 3 - 2018					
100.00 (Ref.: 0.00-99.99)	0.626	0.722	-	0.283	-
Quarter 3 - 2019					
100.00 (Ref.: 0.00-99.99)	0.672	0.290	-	0.514	-
Quarter 3 - 2020					
100.00 (Ref.: 0.00-99.99)	1.036	0.242	0.412	0.230	0.180

Table H.18 Bias Estimates of Prop. of High-Educated Employees by Quarter and Weighting Strategy, BHP 2018-2020

Variable	Unadjusted	Current	COVID-19	Admin	All
Quarter 1 - 2018					
0.00	0.340	0.319	-	0.124	-
0.01 - 14.99	0.095	0.022	-	0.027	-
≥15.00	0.244	0.298	-	0.098	-
Quarter 1 - 2019					
0.00	0.215	0.112	-	0.193	-
0.01 - 14.99	0.159	0.038	-	0.175	-
≥15.00	0.055	0.074	-	0.368	-
Quarter 1 - 2020					
0.00	2.799	2.238	2.159	1.062	1.035
0.01 - 14.99	0.696	0.115	0.138	0.250	0.290
≥15.00	2.103	2.124	2.020	0.812	0.744
Quarter 2 - 2018					
0.00	0.941	0.628	-	0.372	-
0.01 - 14.99	0.204	0.003	-	0.090	-
≥15.00	0.738	0.625	-	0.282	-
Quarter 2 - 2019					
0.00	0.994	0.675	-	0.466	-
0.01 - 14.99	0.260	0.055	-	0.129	-
≥15.00	0.734	0.730	-	0.337	-
Quarter 2 - 2020					
0.00	4.896	3.256	3.174	1.861	1.855
0.01 - 14.99	1.652	0.276	0.266	0.439	0.422
≥15.00	3.243	2.980	2.908	1.422	1.433
Quarter 3 - 2018					
0.00	0.469	0.393	-	0.337	-
0.01 - 14.99	0.368	0.449	-	0.266	-
≥15.00	0.837	0.842	-	0.603	-
Quarter 3 - 2019					
0.00	1.434	0.944	-	0.848	-
0.01 - 14.99	0.788	0.318	-	0.360	-
≥15.00	0.646	0.626	-	0.488	-
Quarter 3 - 2020					
0.00	4.619	2.791	2.843	1.850	1.865
0.01 - 14.99	1.552	0.203	0.196	0.277	0.246
≥15.00	3.067	2.588	2.647	1.574	1.620

Table H.19 Bias Estimates of Prop. of Low-Educated Employees by Quarter and Weighting Strategy, BHP 2018-2020

Variable	Unadjusted	Current	COVID-19	Admin	All
Quarter 1 - 2018					
0.00	0.240	0.152	-	0.120	-
0.01 - 11.99	0.201	0.135	-	0.086	-
≥12.00	0.039	0.017	-	0.034	-
Quarter 1 - 2019					
0.00	0.972	1.098	-	0.721	-
0.01 - 11.99	0.150	0.005	-	0.010	-
≥12.00	1.122	1.102	-	0.731	-
Quarter 1 - 2020					
0.00	0.769	0.004	0.022	0.125	0.111
0.01 - 11.99	0.968	0.363	0.340	0.347	0.325
≥12.00	0.199	0.367	0.319	0.223	0.215
Quarter 2 - 2018					
0.00	0.227	0.090	-	0.023	-
0.01 - 11.99	0.154	0.064	-	0.023	-
≥12.00	0.074	0.154	-	0.046	-
Quarter 2 - 2019					
0.00	0.146	0.484	-	0.113	-
0.01 - 11.99	0.323	0.036	-	0.102	-
≥12.00	0.470	0.519	-	0.011	-
Quarter 2 - 2020					
0.00	1.565	0.110	0.019	0.207	0.270
0.01 - 11.99	1.543	0.144	0.121	0.171	0.169
≥12.00	0.022	0.254	0.102	0.036	0.101
Quarter 3 - 2018					
0.00	0.633	0.694	-	0.102	-
0.01 - 11.99	0.291	0.211	-	0.117	-
≥12.00	0.925	0.905	-	0.015	-
Quarter 3 - 2019					
0.00	0.499	0.008	-	0.276	-
0.01 - 11.99	0.589	0.137	-	0.005	-
≥12.00	0.090	0.145	-	0.281	-
Quarter 3 - 2020					
0.00	1.502	0.004	0.222	0.157	0.059
0.01 - 11.99	1.689	0.337	0.341	0.190	0.187
≥12.00	0.187	0.333	0.563	0.032	0.128

Table H.20 Bias Estimates of Prop. of Marginal Contracts by Quarter and Weighting Strategy, BHP 2018-2020

Variable	Unadjusted	Current	COVID-19	Admin	All
Quarter 1 - 2018					
0.00	0.679	0.743	-	0.271	-
0.00-14.99	0.034	0.012	-	0.045	-
≥15.00	0.713	0.732	-	0.226	-
Quarter 1 - 2019					
0.00	0.156	0.180	-	0.432	-
0.00-14.99	0.165	0.060	-	0.165	-
≥15.00	0.009	0.119	-	0.267	-
Quarter 1 - 2020					
0.00	0.204	0.528	0.605	0.146	0.128
0.00-14.99	0.832	0.277	0.280	0.021	0.014
≥15.00	1.037	0.805	0.885	0.125	0.114
Quarter 2 - 2018					
0.00	0.355	0.424	-	0.028	-
0.00-14.99	0.371	0.209	-	0.342	-
≥15.00	0.726	0.633	-	0.315	-
Quarter 2 - 2019					
0.00	0.344	0.211	-	0.096	-
0.00-14.99	0.657	0.451	-	0.277	-
≥15.00	0.313	0.240	-	0.181	-
Quarter 2 - 2020					
0.00	0.147	0.672	0.740	0.110	0.036
0.00-14.99	1.605	0.477	0.437	0.189	0.203
≥15.00	1.457	1.150	1.178	0.079	0.167
Quarter 3 - 2018					
0.00	0.818	0.845	-	0.027	-
0.00-14.99	0.128	0.060	-	0.031	-
≥15.00	0.946	0.905	-	0.004	-
Quarter 3 - 2019					
0.00	0.890	0.561	-	0.134	-
0.00-14.99	0.539	0.193	-	0.011	-
≥15.00	0.351	0.368	-	0.122	-
Quarter 3 - 2020					
0.00	0.244	0.440	0.622	0.049	0.124
0.00-14.99	1.659	0.485	0.539	0.012	0.038
≥15.00	1.416	0.924	1.161	0.061	0.161

Table H.21 Bias Estimates of Prop. of Mid-Educated Employees by Quarter and Weighting Strategy, BHP 2018-2020

Variable	Unadjusted	Current	COVID-19	Admin	All
Quarter 1 - 2018					
0.00 - 50.45	0.211	0.239	-	0.141	-
50.46 - 72.79	0.375	0.355	-	0.398	-
72.80 - 86.66	0.599	0.543	-	0.607	-
>86.67	0.435	0.428	-	0.067	-
Quarter 1 - 2019					
0.00 - 50.45	0.023	0.089	-	0.318	-
50.46 - 72.79	0.097	0.139	-	0.084	-
72.80 - 86.66	0.442	0.480	-	0.377	-
>86.67	0.516	0.530	-	0.025	-
Quarter 1 - 2020					
0.00 - 50.45	0.540	0.823	0.842	0.108	0.064
50.46 - 72.79	0.892	0.599	0.610	0.414	0.437
72.80 - 86.66	0.552	0.254	0.225	0.140	0.126
>86.67	1.984	1.675	1.677	0.446	0.498
Quarter 2 - 2018					
0.00 - 50.45	0.996	0.997	-	0.417	-
50.46 - 72.79	0.536	0.537	-	0.698	-
72.80 - 86.66	0.089	0.160	-	0.013	-
>86.67	0.370	0.299	-	0.269	-
Quarter 2 - 2019					
0.00 - 50.45	0.059	0.170	-	0.098	-
50.46 - 72.79	0.009	0.120	-	0.176	-
72.80 - 86.66	0.260	0.442	-	0.435	-
>86.67	0.210	0.392	-	0.512	-
Quarter 2 - 2020					
0.00 - 50.45	1.007	1.435	1.465	0.002	0.061
50.46 - 72.79	0.701	0.173	0.266	0.359	0.298
72.80 - 86.66	0.665	0.067	0.021	0.120	0.177
>86.67	2.372	1.674	1.709	0.481	0.414
Quarter 3 - 2018					
0.00 - 50.45	0.065	0.133	-	0.093	-
50.46 - 72.79	0.644	0.647	-	0.406	-
72.80 - 86.66	0.193	0.148	-	0.296	-
>86.67	0.386	0.366	-	0.204	-
Quarter 3 - 2019					
0.00 - 50.45	0.659	0.443	-	0.694	-
50.46 - 72.79	0.526	0.304	-	0.195	-
72.80 - 86.66	0.285	0.033	-	0.182	-
>86.67	0.153	0.105	-	0.682	-
Quarter 3 - 2020					
0.00 - 50.45	0.343	0.517	0.432	0.230	0.287
50.46 - 72.79	0.496	0.048	0.078	0.581	0.675
72.80 - 86.66	0.926	0.399	0.404	0.031	0.021
>86.67	1.765	0.964	0.758	0.780	0.941

Table H.22 Bias Estimates of Prop. of Part-Time Contracts by Quarter and Weighting Strategy, BHP 2018-2020

Variable	Unadjusted	Current	COVID-19	Admin	All
Quarter 1 - 2018					
0.00	0.119	0.123	-	0.285	-
0.01 - 19.99	0.299	0.319	-	0.254	-
≥20.00	0.180	0.196	-	0.031	-
Quarter 1 - 2019					
0.00	0.912	0.890	-	0.521	-
0.01 - 19.99	0.150	0.038	-	0.182	-
≥20.00	0.762	0.852	-	0.339	-
Quarter 1 - 2020					
0.00	0.028	0.251	0.258	0.020	0.009
0.01 - 19.99	0.834	0.232	0.249	0.346	0.348
≥20.00	0.806	0.483	0.506	0.366	0.340
Quarter 2 - 2018					
0.00	0.128	0.216	-	0.485	-
0.01 - 19.99	0.417	0.313	-	0.407	-
≥20.00	0.545	0.529	-	0.892	-
Quarter 2 - 2019					
0.00	0.737	0.517	-	0.258	-
0.01 - 19.99	0.158	0.035	-	0.002	-
≥20.00	0.579	0.482	-	0.256	-
Quarter 2 - 2020					
0.00	0.087	0.883	0.936	0.185	0.282
0.01 - 19.99	1.830	0.422	0.457	0.052	0.065
≥20.00	1.743	1.305	1.393	0.237	0.348
Quarter 3 - 2018					
0.00	0.164	0.145	-	0.209	-
0.01 - 19.99	0.474	0.594	-	0.423	-
≥20.00	0.310	0.448	-	0.214	-
Quarter 3 - 2019					
0.00	0.632	0.324	-	0.101	-
0.01 - 19.99	0.302	0.049	-	0.112	-
≥20.00	0.329	0.373	-	0.212	-
Quarter 3 - 2020					
0.00	0.618	0.323	0.503	0.202	0.118
0.01 - 19.99	1.711	0.369	0.211	0.068	0.081
≥20.00	1.093	0.692	0.713	0.134	0.199

Table H.23 Bias Estimates of Prop. of Regular Contracts by Quarter and Weighting Strategy, BHP 2018-2020

Variable	Unadjusted	Current	COVID-19	Admin	All
Quarter 1 - 2018					
0.00 - 70.73	1.148	1.181	-	0.757	-
70.74 - 87.57	0.757	0.729	-	0.949	-
87.58 - 96.93	0.080	0.125	-	0.192	-
>96.94	0.471	0.577	-	0.000	-
Quarter 1 - 2019					
0.00 - 70.73	0.009	0.081	-	0.119	-
70.74 - 87.57	0.295	0.302	-	0.385	-
87.58 - 96.93	0.083	0.014	-	0.021	-
>96.94	0.221	0.235	-	0.287	-
Quarter 1 - 2020					
0.00 - 70.73	1.312	1.010	1.122	0.445	0.502
70.74 - 87.57	0.598	0.249	0.253	0.410	0.426
87.58 - 96.93	0.775	0.420	0.417	0.239	0.229
>96.94	0.061	0.341	0.452	0.204	0.153
Quarter 2 - 2018					
0.00 - 70.73	0.025	0.202	-	0.208	-
70.74 - 87.57	0.699	0.776	-	0.420	-
87.58 - 96.93	0.213	0.053	-	0.025	-
>96.94	0.461	0.521	-	0.237	-
Quarter 2 - 2019					
0.00 - 70.73	0.198	0.202	-	0.375	-
70.74 - 87.57	0.031	0.078	-	0.343	-
87.58 - 96.93	0.454	0.312	-	0.197	-
>96.94	0.621	0.437	-	0.228	-
Quarter 2 - 2020					
0.00 - 70.73	1.726	1.141	1.107	0.324	0.356
70.74 - 87.57	0.873	0.175	0.073	0.468	0.501
87.58 - 96.93	1.050	0.267	0.262	0.096	0.103
>96.94	0.196	0.699	0.772	0.695	0.754
Quarter 3 - 2018					
0.00 - 70.73	1.192	1.117	-	0.343	-
70.74 - 87.57	0.167	0.134	-	0.292	-
87.58 - 96.93	0.265	0.207	-	0.099	-
>96.94	0.760	0.776	-	0.048	-
Quarter 3 - 2019					
0.00 - 70.73	0.053	0.097	-	0.069	-
70.74 - 87.57	0.427	0.277	-	0.161	-
87.58 - 96.93	0.412	0.161	-	0.140	-
>96.94	0.892	0.535	-	0.048	-
Quarter 3 - 2020					
0.00 - 70.73	2.427	1.633	1.809	1.135	1.165
70.74 - 87.57	1.755	1.105	1.082	0.444	0.400
87.58 - 96.93	1.135	0.305	0.349	0.153	0.165
>96.94	0.463	0.223	0.379	0.538	0.600

Table H.24 Bias Estimates of Prop. of Unknown-Educated Employees by Quarter and Weighting Strategy, BHP 2018-2020

Variable	Unadjusted	Current	COVID-19	Admin	All
Quarter 1 - 2018					
0.00 (Ref.: 0.01-100.00)	0.085	0.021	-	0.029	-
Quarter 1 - 2019					
0.00 (Ref.: 0.01-100.00)	0.410	0.458	-	0.332	-
Quarter 1 - 2020					
0.00 (Ref.: 0.01-100.00)	0.357	0.079	0.040	0.061	0.121
Quarter 2 - 2018					
0.00 (Ref.: 0.01-100.00)	0.262	0.317	-	0.335	-
Quarter 2 - 2019					
0.00 (Ref.: 0.01-100.00)	0.426	0.619	-	0.215	-
Quarter 2 - 2020					
0.00 (Ref.: 0.01-100.00)	0.496	0.242	0.232	0.141	0.098
Quarter 3 - 2018					
0.00 (Ref.: 0.01-100.00)	0.023	0.036	-	0.719	-
Quarter 3 - 2019					
0.00 (Ref.: 0.01-100.00)	0.404	0.669	-	0.319	-
Quarter 3 - 2020					
0.00 (Ref.: 0.01-100.00)	0.364	1.256	1.481	0.537	0.686

Table H.25 Bias Estimates of Quartile of Wage Distribution by Quarter and Weighting Strategy, BHP 2018-2020

Variable	Unadjusted	Current	COVID-19	Admin	All
Quarter 1 - 2018					
First Quartile	0.419	0.451	-	0.205	-
Second Quartile	0.269	0.272	-	0.301	-
Third Quartile	0.160	0.160	-	0.020	-
Fourth Quartile	0.748	0.707	-	0.485	-
Missings	0.758	0.688	-	0.602	-
Quarter 1 - 2019					
First Quartile	0.731	0.689	-	0.582	-
Second Quartile	0.045	0.021	-	0.058	-
Third Quartile	0.077	0.023	-	0.139	-
Fourth Quartile	0.137	0.026	-	0.148	-
Missings	0.561	0.661	-	0.237	-
Quarter 1 - 2020					
First Quartile	0.352	0.721	0.877	0.758	0.859
Second Quartile	0.331	0.260	0.263	0.308	0.295
Third Quartile	0.564	0.988	1.036	1.115	1.136
Fourth Quartile	1.356	0.910	0.823	0.162	0.108
Missings	1.474	0.903	0.926	0.113	0.125
Quarter 2 - 2018					
First Quartile	0.330	0.169	-	0.089	-
Second Quartile	0.084	0.066	-	0.191	-
Third Quartile	0.009	0.044	-	0.053	-
Fourth Quartile	0.573	0.262	-	0.203	-
Missings	0.169	0.072	-	0.358	-
Quarter 2 - 2019					
First Quartile	0.163	0.168	-	0.161	-
Second Quartile	0.318	0.231	-	0.238	-
Third Quartile	0.478	0.445	-	0.325	-
Fourth Quartile	0.752	0.596	-	0.399	-
Missings	0.748	0.642	-	0.647	-
Quarter 2 - 2020					
First Quartile	0.501	0.032	0.105	0.210	0.258
Second Quartile	0.232	0.356	0.280	0.410	0.355
Third Quartile	1.256	0.810	0.719	0.080	0.056
Fourth Quartile	2.592	1.518	1.333	0.301	0.205
Missings	3.115	1.940	1.877	0.181	0.163
Quarter 3 - 2018					
First Quartile	0.122	0.045	-	0.034	-
Second Quartile	0.599	0.600	-	0.354	-
Third Quartile	0.127	0.050	-	0.226	-
Fourth Quartile	0.611	0.451	-	0.239	-
Missings	0.018	0.144	-	0.145	-
Quarter 3 - 2019					
First Quartile	0.701	0.647	-	0.541	-
Second Quartile	0.108	0.143	-	0.004	-
Third Quartile	0.521	0.406	-	0.323	-
Fourth Quartile	0.493	0.233	-	0.193	-
Missings	0.421	0.135	-	0.021	-
Quarter 3 - 2020					
First Quartile	0.925	0.545	0.432	0.004	0.007
Second Quartile	0.166	0.451	0.422	0.415	0.352
Third Quartile	0.275	0.085	0.149	1.004	0.999
Fourth Quartile	3.606	2.350	2.369	1.041	1.154
Missings	3.123	2.171	2.211	0.447	0.500

Table H.26 Bias Estimates of Short-Time Work Dummy by Quarter and Weighting Strategy, BHP 2018-2020

Variable	Unadjusted	Current	COVID-19	Admin	All
Quarter 1 - 2020					
No Short-Time Work (Ref.: Short-Time Work)	0.931	0.881	0.747	0.883	0.777
Quarter 2 - 2020					
No Short-Time Work (Ref.: Short-Time Work)	0.209	0.371	0.117	0.108	0.059
Quarter 3 - 2020					
No Short-Time Work (Ref.: Short-Time Work)	1.256	0.837	0.843	0.902	0.914

H.4 Model Fit Statistics

Table H.27 Model Fit Statistics, by Quarter and Weighting Strategy, BHP 2018 - 2020

Weighting Scheme	adj. McFadden R^2			AIC			BIC		
	Q1	Q2	Q3	Q1	Q2	Q3	Q1	Q2	Q3
2018									
Current	0.006	0.016	0.014	150.98	151.18	112.18	1,396,020	1,387,313	970,839
COVID-19	-	-	-	-	-	-	-	-	-
Admin	0.044	0.055	0.098	145.16	145.19	102.71	1,339,066	1,329,135	882,014
All	-	-	-	-	-	-	-	-	-
2019									
Current	0.006	0.014	0.015	139.00	158.81	158.10	1,264,581	1,483,651	1,461,961
COVID-19	-	-	-	-	-	-	-	-	-
Admin	0.047	0.041	0.038	133.29	154.52	154.40	1,209,276	1,441,449	1,426,054
All	-	-	-	-	-	-	-	-	-
2020									
Current	0.008	0.011	0.012	206.58	204.91	207.84	2,104,169	2,564,482	2,538,286
COVID-19	0.012	0.014	0.015	205.75	204.36	207.22	2,095,402	2,557,434	2,530,372
Admin	0.028	0.029	0.032	202.33	201.18	203.49	2,059,238	2,515,931	2,483,003
All	0.037	0.037	0.036	200.52	199.53	202.74	2,040,052	2,494,453	2,473,513

H.5 Response Propensity Models

Table H.28 Response Propensity Model I by Quarter, BHP 2020

	(1) Quarter 1		(2) Quarter 2		(3) Quarter 3	
DV: Response						
Industry (Ref.: Agriculture)						
Bergbau/Energie/Wasser/Abfall	1.085	(0.287)	1.138	(0.244)	0.913	(0.197)
Verarbeitendes Gewerbe	1.207	(0.297)	1.256	(0.252)	1.072	(0.218)
Baugewerbe	1.480	(0.415)	0.822	(0.185)	0.645	(0.146)
Handel und KFZ-Reparatur	1.070	(0.307)	1.144	(0.269)	0.907	(0.218)
Verkehr und Lagerei	0.822	(0.228)	0.830	(0.186)	0.792	(0.182)
Information und Kommunikation	0.762	(0.244)	0.961	(0.243)	0.911	(0.237)
Finanz- und Versicherungsdienstleistungen	0.839	(0.287)	1.134	(0.306)	1.177	(0.326)
Unternehmensnahe Dienstleistungen	0.968	(0.262)	1.010	(0.222)	1.059	(0.234)
Sonstige Dienstleistungen	1.051	(0.272)	1.094	(0.231)	0.965	(0.206)
Öffentliche Verwaltung/Sozialversicherung	2.031*	(0.625)	1.682*	(0.409)	1.467	(0.362)
Number of Employees (Ref.:1-9)						
10-19	1.005	(0.179)	1.091	(0.146)	0.908	(0.127)
20-49	0.884	(0.164)	0.986	(0.142)	0.985	(0.150)
50-249	1.062	(0.255)	0.916	(0.169)	1.053	(0.205)
≥250	0.748	(0.207)	0.658*	(0.140)	0.578*	(0.125)
Earliest 50% of Response in Q4 (Ref.: Latest 50%)						
First 50%	1.295*	(0.134)	1.284**	(0.106)	1.089	(0.093)
Decade of Foundation (Ref.: 70s/80s)						
90s	0.914	(0.146)	0.998	(0.126)	0.822	(0.106)
00s	1.111	(0.173)	1.086	(0.135)	0.947	(0.121)
10s	1.034	(0.167)	0.985	(0.125)	0.750*	(0.097)
Avg. Age of Employees (Ref.: 0.00 - 38.99)						
39.00-43.49	0.982	(0.154)	1.014	(0.126)	0.944	(0.121)
43.50-47.99	1.187	(0.200)	1.062	(0.136)	0.973	(0.128)
≥48.00	1.110	(0.182)	1.242	(0.159)	1.098	(0.143)
Proportion of Female Employees (Ref.: 0.00 - 18.18)						
18.19 - 41.93	1.273	(0.200)	1.054	(0.133)	1.072	(0.138)
41.94 - 68.38	1.168	(0.204)	0.902	(0.125)	0.952	(0.136)
>68.39	0.973	(0.170)	0.671**	(0.094)	0.720*	(0.101)
Proportion of Fixed-Term Employees (Ref.: 0.00)						
0.01 - 15.99	1.003	(0.147)	1.129	(0.130)	1.111	(0.139)
≥16.00	0.822	(0.124)	1.157	(0.133)	0.998	(0.116)
Proportion of Apprentices (Ref.: 0.00)						
0.01-100	1.142	(0.185)	1.187	(0.148)	1.330*	(0.172)
Proportion of Full-Time Employees (Ref.: 0.00 - 34.40)						
34.41 - 65.35	1.104	(0.191)	1.357*	(0.179)	1.183	(0.163)
65.36 - 85.15	0.866	(0.194)	1.337	(0.235)	1.313	(0.238)
>85.16	1.025	(0.285)	1.057	(0.231)	1.159	(0.263)

Table H.29 Response Propensity Model II by Quarter, BHP 2020

	(1) Quarter 1		(2) Quarter 2		(3) Quarter 3	
DV: Response						
Proportion of Part-Time Employees (Ref.: 0.00)						
0.01 - 19.99	1.034	(0.179)	0.954	(0.130)	1.013	(0.143)
≥20.00	0.880	(0.168)	0.972	(0.146)	1.085	(0.166)
Proportion of German Employees (Ref.: 100.00)						
0.00-99.99	0.814	(0.107)	0.963	(0.097)	0.959	(0.098)
Proportion of Regular Employees (Ref.: 0.00 - 70.73)						
70.74 - 87.57	1.288	(0.218)	0.994	(0.129)	1.203	(0.163)
87.58 - 96.93	1.566*	(0.344)	1.128	(0.198)	1.270	(0.232)
>96.94	1.111	(0.300)	1.392	(0.278)	1.356	(0.278)
Proportion of Marginal Employees (Ref.: 0.00)						
0.01-14.99	0.869	(0.174)	1.139	(0.169)	1.038	(0.164)
≥15.00	0.827	(0.204)	1.122	(0.199)	1.149	(0.211)
Proportion of Mid-Educated Employees (Ref.: 0.00 - 50.45)						
50.46 - 72.79	1.121	(0.187)	0.933	(0.122)	0.974	(0.129)
72.80 - 86.66	1.011	(0.186)	0.982	(0.143)	1.090	(0.167)
>86.67	0.846	(0.176)	1.080	(0.183)	1.237	(0.217)
Proportion of Low-Educated Employees (Ref.: 0.00)						
0.01 - 11.99	1.164	(0.204)	1.033	(0.138)	1.043	(0.141)
≥12.00	0.972	(0.176)	1.074	(0.148)	1.074	(0.153)
Proportion of High-Educated Employees (Ref.: 0.00)						
0.01 - 14.99	1.311	(0.194)	1.357*	(0.161)	1.269	(0.162)
≥15.00	1.560*	(0.284)	1.572**	(0.229)	1.577**	(0.239)
Proportion of High-Educated Employees (Ref.: First Quartile)						
Second Quartile	0.861	(0.157)	0.930	(0.131)	1.056	(0.155)
Third Quartile	0.625*	(0.118)	0.971	(0.140)	0.909	(0.136)
Fourth Quartile	0.830	(0.169)	1.021	(0.156)	1.245	(0.198)
Missings	0.807	(0.163)	0.929	(0.150)	0.914	(0.155)
Establishment Foundation (Ref.: Founded Before Sampling Year)						
Founded in Sampling Year	0.933	(0.305)	1.107	(0.281)	0.844	(0.235)
West Germany (Ref.: East Germany)						
West Germany	1.080	(0.152)	1.178	(0.125)	1.244*	(0.137)
Constant	2.651*	(1.182)	0.605	(0.210)	0.805	(0.287)
Observations	10664		13122		12794	

Notes: Odds-Ratios; Standard errors in parentheses

* $p<0.05$, ** $p<0.01$, *** $p<0.001$
